# Moving towards the use of artificial intelligence in pain management

**DOI:** 10.1002/ejp.4748

**Published:** 2024-11-10

**Authors:** Ryan Antel, Sera Whitelaw, Genevieve Gore, Pablo Ingelmo

**Affiliations:** ^1^ Department of Anesthesia McGill University Montreal Quebec Canada; ^2^ Faculty of Medicine and Health Sciences McGill University Montreal Quebec Canada; ^3^ Schulich Library of Physical Sciences, Life Sciences, and Engineering McGill University Montreal Quebec Canada; ^4^ Edwards Family Interdisciplinary Center for Complex Pain, Montreal Children's Hospital McGill University Health Center Montreal Quebec Canada; ^5^ Alan Edwards Center for Research in Pain Montreal Quebec Canada; ^6^ Research Institute McGill University Health Center Montreal Quebec Canada

## Abstract

**Background and Objective:**

While the development of artificial intelligence (AI) technologies in medicine has been significant, their application to acute and chronic pain management has not been well characterized. This systematic review aims to provide an overview of the current state of AI in acute and chronic pain management.

**Databases and Data Treatment:**

This review was registered with PROSPERO (ID# CRD42022307017), the international registry for systematic reviews. The search strategy was prepared by a librarian and run in four electronic databases (Embase, Medline, Central, and Web of Science). Collected articles were screened by two reviewers. Included studies described the use of AI for acute and chronic pain management.

**Results:**

From the 17,601 records identified in the initial search, 197 were included in this review. Identified applications of AI were described for treatment planning as well as treatment delivery. Described uses include prediction of pain, forecasting of individualized responses to treatment, treatment regimen tailoring, image‐guidance for procedural interventions and self‐management tools. Multiple domains of AI were used including machine learning, computer vision, fuzzy logic, natural language processing and expert systems.

**Conclusion:**

There is growing literature regarding applications of AI for pain management, and their clinical use holds potential for improving patient outcomes. However, multiple barriers to their clinical integration remain including lack validation of such applications in diverse patient populations, missing infrastructure to support these tools and limited provider understanding of AI.

**Significance:**

This review characterizes current applications of AI for pain management and discusses barriers to their clinical integration. Our findings support continuing efforts directed towards establishing comprehensive systems that integrate AI throughout the patient care continuum.

## 1 INTRODUCTION

The International Association for the Study of Pain defines pain as ‘an unpleasant sensory and emotional experience associated with, or resembling that associated with, actual or potential tissue damage’ (Raja et al., 2020, 2017). The perception of pain can have diverse and profound impacts on individuals, with life‐changing negative impacts on quality of living (Duenas et al., 2016; Froud et al., 2014; Michaelides & Zis, 2019). However, the interpretation of such experience is influenced to varying degrees by physiological, psychological, and social factors as described by the widely adopted biopsychosocial model of pain (Meints & Edwards, 2018). Unrelieved pain in the acute setting has consequences beyond the immediate experience, including reduced quality of life, impaired sleep and physical function, and increased risk of developing chronic pain (Sinatra, 2010). On the other hand, chronic pain is often described as a disease in its own right; affecting not only patients, but their families and friends as well (Vega et al., 2018). The true prevalence of pain is difficult to characterize largely due to the subjective nature of symptoms and a lack of consensus regarding specific diagnoses and definitions of conditions. However, it is estimated that pain affects billions of people globally on a daily basis (Zimmer et al., 2022). The annual cost of diagnosing and managing pain in the United States is greater than the annual costs of heart disease ($309 billion), cancer ($243 billion), and diabetes ($188 billion) (Henschke et al., 2015).

Promising areas of research regarding the effective management of patients experiencing acute and chronic pain are significant, including the study of predictive modelling and precision medicine approaches (Cohen et al., 2021). The use of large data collections for predictive modelling and precision medicine has recently been emphasized in the literature, including the use of computational models to process and mine data collections, develop diagnostic and prognostic models, and predict response to potential treatments (König et al., 2017). For instance Niculescu et al. successfully identified objective blood biomarkers for pain using genetic expression data to allow improved diagnostics and targeted therapeutics (Niculescu et al., 2019). Similarly Lee et al. used magnetic resonance imaging to identify neural signatures associated with pain to classify treatment responders and identify therapeutic targets (Lee, Wei, et al., 2021). A recent review by Edwards et al. describes precision medicine approaches for conducting clinical trials on chronic pain using clinical patient data (Edwards et al., 2023). All of these approaches aim to use collected data to develop clinically relevant models, with the objective of applying these analyses to then inform further assessment and treatment of patients (Subramanian et al., 2020).

Artificial intelligence (AI) is broadly described as the use of algorithms to give machines the ability to reason and perform functions such as problem‐solving, object and word recognition, inference of world states, and decision‐making (Bellman, 1978). This is achieved through the use of multiple distinct technologies, all of which can be considered branches of AI. These technologies include machine learning, computer vision, natural language processing (NLP), expert systems and fuzzy logic (Chen & Decary, 2020). Table 1 provides a basic overview of these techniques, and an informative in‐depth discussion can be found in a recent review published by Chen et al. (Chen & Decary, 2020). Another recent review by Hagedorn et al. describes multiple novel applications of AI within pain medicine such as clinical trial optimization using machine learning, streamlining physician workflow and doctor‐patient communication using NLP, and analysing patient outcomes using deep learning (Hagedorn et al., 2024). However, the use of AI also has the potential to revolutionize our precision‐medicine approach to managing patients with acute and chronic pain.

**TABLE 1:** Overview of commonly applied artificial intelligence technologies.

Machine learning	Machine learning refers to the use of mathematical algorithms to identify patterns in very large datasets (Hashimoto et al., 2020; Rowe, 2019). Different learning methods can be employed to perform distinctive tasks including supervised learning, unsupervised learning and reinforcement learning.
Supervised learning	Supervised learning draws knowledge from previously curated training datasets to make further predictions from future datasets (Gareth James, 2013; Hastie et al., 2009). To describe this using an analogy, a supervised learning algorithm can be thought of as a student whereas a collection of labelled data can be thought of as a teacher. The labelled data (teacher) provides the supervised learning algorithm (student) with examples of correct answers (output labels) along with corresponding input data (i.e. a math problem with the associated correct solutions). The supervised learning algorithm can then ‘learn’ from this associated data to infer the solutions to different (yet similar) problems in the future.
Unsupervised learning	Unsupervised learning aims to categorize individual instances in a dataset into distinct categories determined by the algorithm, without being informed by a previously created training dataset (Gareth James, 2013; Hastie et al., 2009). This is in contrast to supervised learning as no associated input–output data is provided to the algorithm. Rather, the unsupervised algorithm aims to discover patterns or groups occurring within a collection of data without being provided with associated output labels.
Reinforcement learning	Reinforcement learning is the technique of training an algorithm for a specific task where no single answer is correct, but an overall outcome is desired. More specifically, reinforcement learning algorithms are able to use ‘trial and error’ and incorporate feedback from its own actions and experiences to maximize the total cumulative reward desired (Choi, Baker, et al., 2020). To describe this using an analogy, we can think of teaching a dog to catch a ball. Rather than teaching this dog how to explicitly catch a ball, we can throw the ball towards the dog and give the dog a treat every time the ball is caught. If the dog fails to catch the ball, then we do not give the dog a treat. By repeating this exercise multiple times, the dog will eventually learn which actions lead to it receiving a treat. Thus, the dog will learn how to catch a ball by maximizing the number of treats it receives (i.e. will maximize reward via ‘trial and error’) to achieve the overall outcome desired.
Computer vision	Computer vision describes a computing system's ability to interpret the visual world in numerical or symbolic form from images, video and other visual data (Hashimoto et al., 2020).
Natural language processing	Natural language processing uses computational techniques to learn, understand, and produce human language content (Hirschberg & Manning, 2015). Natural language processing does not simply imply the recognition of letters and words but entails a deeper understanding of syntax and semantics to extract meaning from language.
Expert systems	Expert systems are a branch of AI that draw from a knowledge base and a set of rules for applying this knowledge base to situations fed to the system (Klar & Zaiss, 1990). This is used to make logical predictions about events taking place in the future or reach a logical conclusion about why an event occurred in the past (Klar & Zaiss, 1990).
Fuzzy logic	Fuzzy logic can be incorporated within frameworks to facilitate AI‐based functions (Hashimoto et al., 2020). Unlike binary logic, where concepts of ‘true’ and ‘false’ are relied upon to reach conclusions, fuzzy logic allows for the inclusion of partial truth or degrees of truth. This permits fuzzy logic systems to handle ambiguous information (Hashimoto et al., 2020).
Boosting: extreme gradient boosting; tradient boosting	Subset of machine learning that manipulates training data by generating a large number of pseudo datasets by resampling the original observations with replacement to reduce variance, resulting in an ensemble of decision trees which are averaged to make the best overall prediction (Klug et al., 2020).
Decision trees	Subset of machine learning that classifies data items by posing a series of questions about features associated with the items to split the dataset into distinct classes. Each split has an edge that connects either to a new decision node that contains another feature to further split the data into homogenous groups or to a terminal node (Choi, Baker, et al., 2020).
Expert system	System containing a knowledge base and inference/rules engine—A set of rules for applying the knowledge base to situations provided to the program. This is used to make a logical prediction about events taking place in the future or reach a logical conclusion about why an event occurred in the past (Holman & Cookson, 1987).
Bayes classifier	Probabilistic classification method based on Bayes' theorem with the assumption of independence between features using training datasets to make predictions (Matsangidou et al., 2021).
K‐Means classifier	Subset of machine learning that divides a number of data points into a number of clusters based on the nearest mean (Matsangidou et al., 2021).
K‐Nearest neighbours	Subset of machine learning that uses the proximity of the data in dataspace to make classifications or predictions about the grouping of an individual data point (Goin, 1984).
Neural networks	Network of nodes that communicate with other nodes via connections that are weighted based upon their ability to provide a desired outcome Choi, Baker, et al., 2020.
Random forest	Subset of machine learning that produces multiple decision trees using a subsample of features to create each decision tree. The majority vote among trees is then used as the model's final class prediction Choi, Baker, et al., 2020.
Support vector machines	Subset of machine learning that classifies data by creating a decision boundary, known as the hyperplane, that is orientated as far as possible from the closest data points from each observed class of data (Noble, 2006).
Regression: linear, logistic, elastic net, lasso	An umbrella term for algorithms that characterize the strength of the relationship between a dependent variable and one or more explanatory variables (Biship, 2007).
K‐means clustering	Clustering method that classifies classify objects into a specified number of groups (*k* groups). Each group is centered around their mean, and the algorithm attempts to minimize the distance between each observation and their corresponding mean (Nedyalkova et al., 2021).
Hierarchical clustering analysis	Clustering method that begins by assuming that each data point is its own cluster. At each sequential step in data clustering, the most similar cluster pairs are combined according to the chosen similarity measure. This process is repeated until predetermined criteria are met (Akman et al., 2019).

As such, this systematic review aims to: (1) Characterize current applications of AI for acute and chronic pain management; (2) discuss current barriers to the implementation of such technologies.

## 2 METHODS

The Preferred Reporting Items for Systematic Reviews and Meta‐Analyses (PRISMA) guidelines for systematic reviews was followed for this review (Page et al., 2021). This systematic review has been registered with PROSPERO (ID # CRD42022307017), the international registry for systematic reviews (Schiavo, 2019).

### 2.1 Identifying relevant studies

A senior medical librarian searched the following databases from inception until October 2023: Embase (Ovid), Medline (Ovid), Central (Cochrane Library), and Web of Science (SCI‐EXPANDED, CPCI‐S, ESCI). The search strategy used variations in text words found in the title, abstract or keyword fields, and relevant subject headings to retrieve articles looking at the use of AI for the management of acute and chronic pain. Various forms of the central terms ‘AI’, ‘pain’ and ‘analgesia’ were used to identify relevant articles. The search strategy had no language restriction. See Data S1 for the full search strategy.

### 2.2 Study selection

**FIGURE 1:**
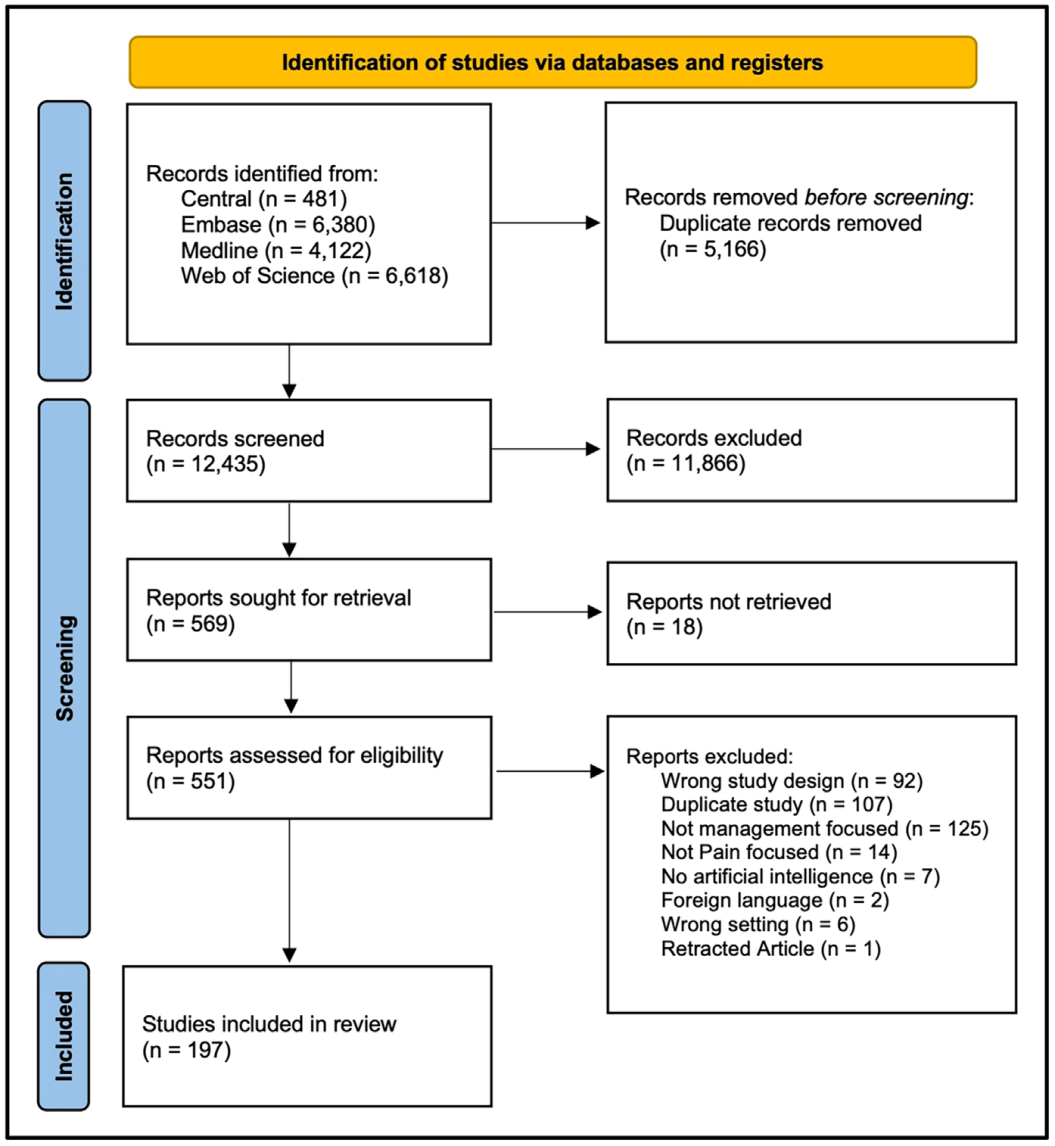
PRISMA flow diagram.

All titles and abstracts obtained in the literature search were manually and independently screened by two authors using Rayyan, an online screening tool (Ouzzani et al., 2016). Identified relevant articles then underwent full‐text screening independently by two authors, with disagreements resolved through discussion. Articles included in the final review described applications of AI that focused on the management of acute and chronic pain in the adult (>18 years old) population. Given significant heterogeneity in participant age reporting practices in the collected literature, we opted to include all studies with a reported mean participant age greater than 18 years old. Articles describing the use of AI solely for the real‐time identification or assessment of pain intensity without further management guidance were excluded and have been extensively reviewed elsewhere (Cascella, Scarpati, et al., 2023). Similarly, as this review intended to focus on the use of AI as it pertains to the treatment of pain, applications meant to diagnose underlying causes of pain were excluded, and have also been broadly summarized elsewhere (D'Antoni et al., 2022). Articles written in languages other than English and French without available translation, as well as articles in the form of review articles, conference abstracts, editorials and commentaries were also excluded. There was no further limitation on study design. A Preferred Reporting Items for Systematic Reviews and Meta‐Analysis (PRISMA) diagram was used to record the screening decisions (Figure 1) (Page et al., 2021).

### 2.3 Data extraction

Following the selection of studies, data from each article was extracted and organized into 17 categories in a standardized data extraction form developed in Microsoft Excel. This was done independently by two authors (RA, SW) to record the information and synthesize it in summary format. Extracted information included author name(s), year of publication, title, location of study, study design, overall study aim, targeted study population, number of included participants, description of discussed AI intervention, domain of AI used, data source(s) used for given AI tool, evaluation(s) of AI tool accuracy/efficacy, main results of study, identified barriers to clinical integration of described application, identified facilitators to clinical integration of described application, chronicity of pain discussed in article (acute versus chronic), and a category for additional pertinent information of interest. See Data S2 for an illustration of the data extraction form used.

### 2.4 Risk of bias assessment, collating, summarizing and reporting results

Information in the data extraction form was collated and the findings and trends as they relate to AI in acute and chronic pain management were recorded and summarized. The risk of bias of included publications was assessed depending on publication type using previously published risk assessment tools. The Template for Intervention Description and Replication tool (TIDieR) (Hoffmann et al., 2014) was used to asses risk of bias of included publications that describe interventions using AI, whereas the Prediction Model Study Risk of Bias Assessment Tool (PROBAST) was applied to studies describing AI prediction models (Wolff et al., 2019). Both of these previously published tools enable researchers to characterize the risk of bias of studies based upon specified categories of potential risk.

## 3 RESULTS

### 3.1 Study characteristics

**FIGURE 2:**
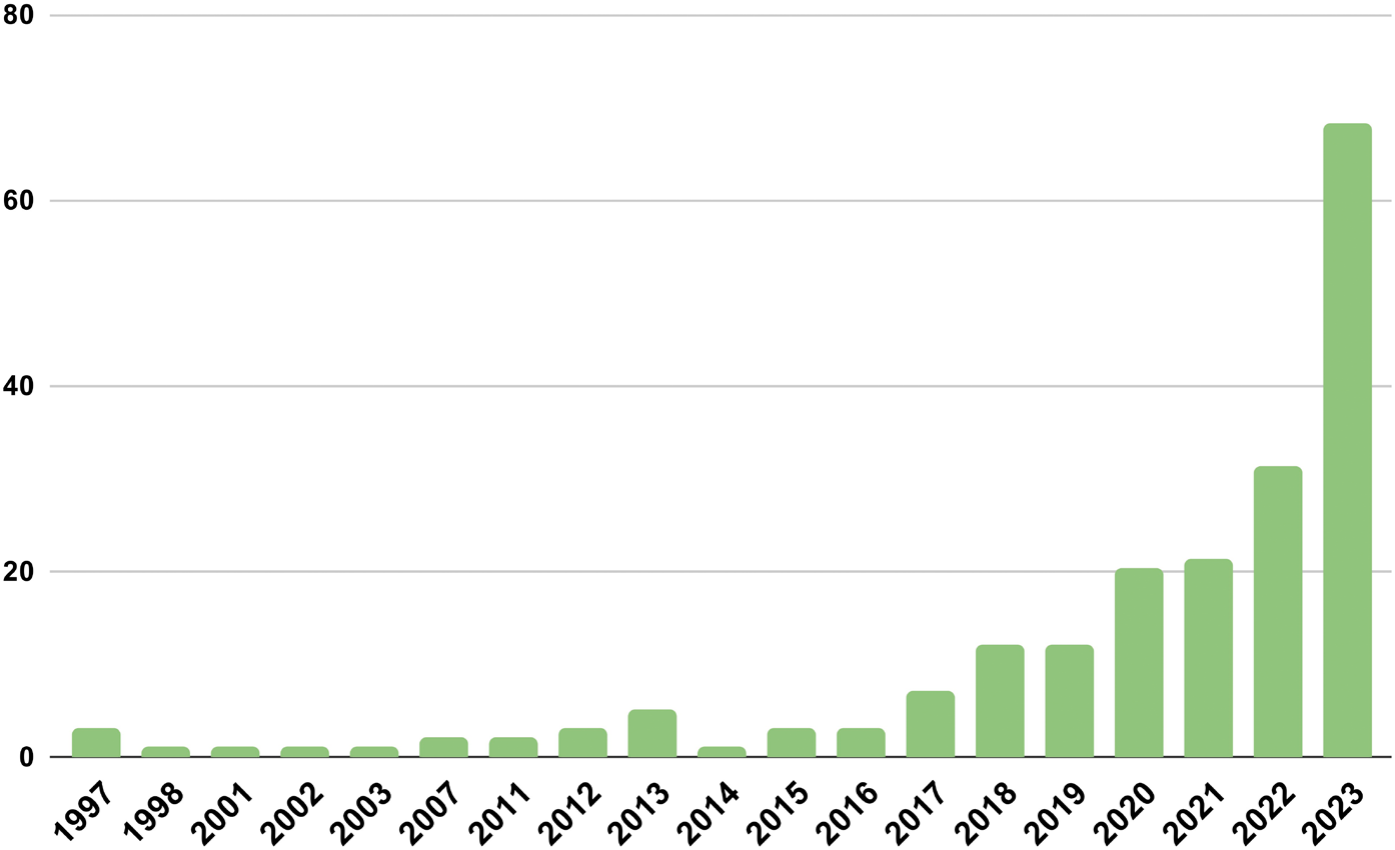
Dates of publication of included articles.

**FIGURE 3:**
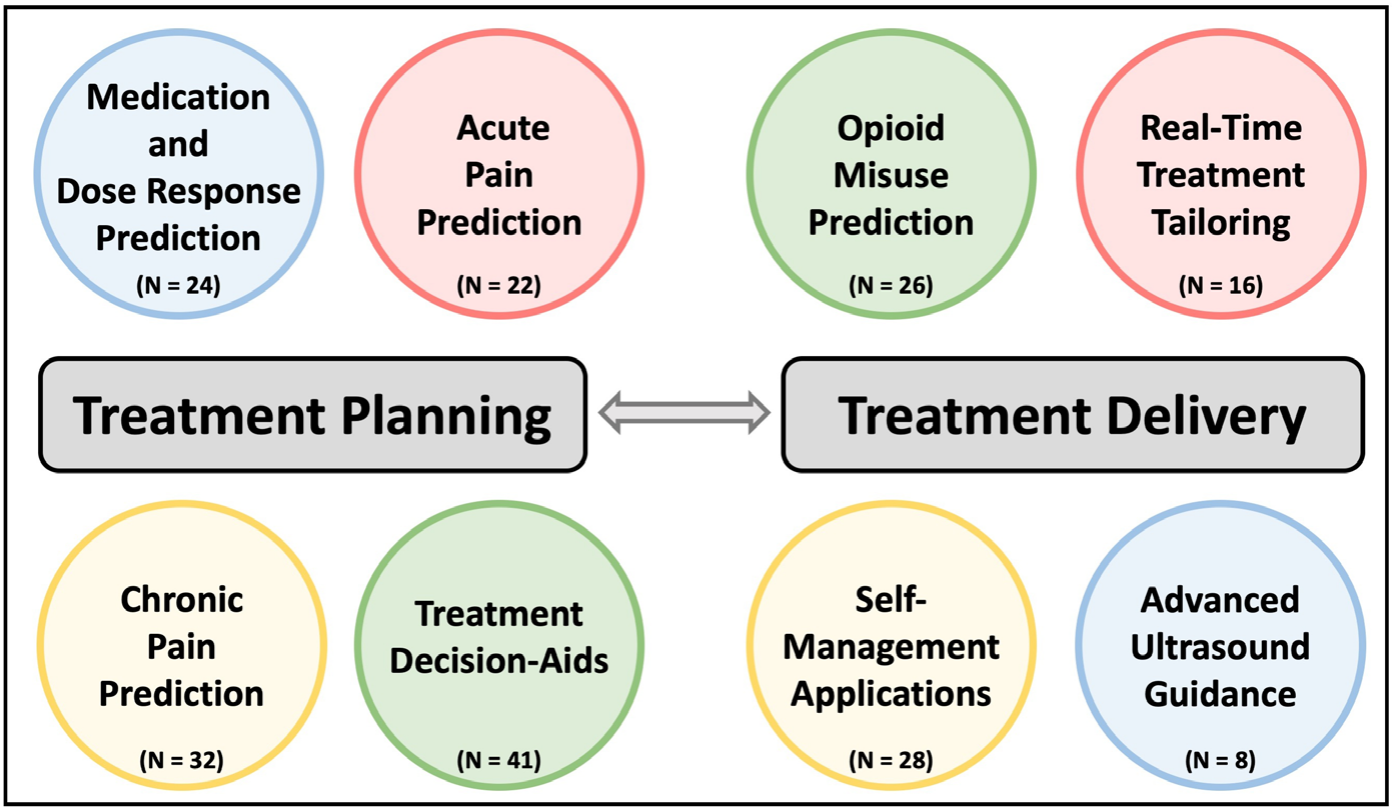
Overview of applications using artificial intelligence for pain management.

**FIGURE 4:**
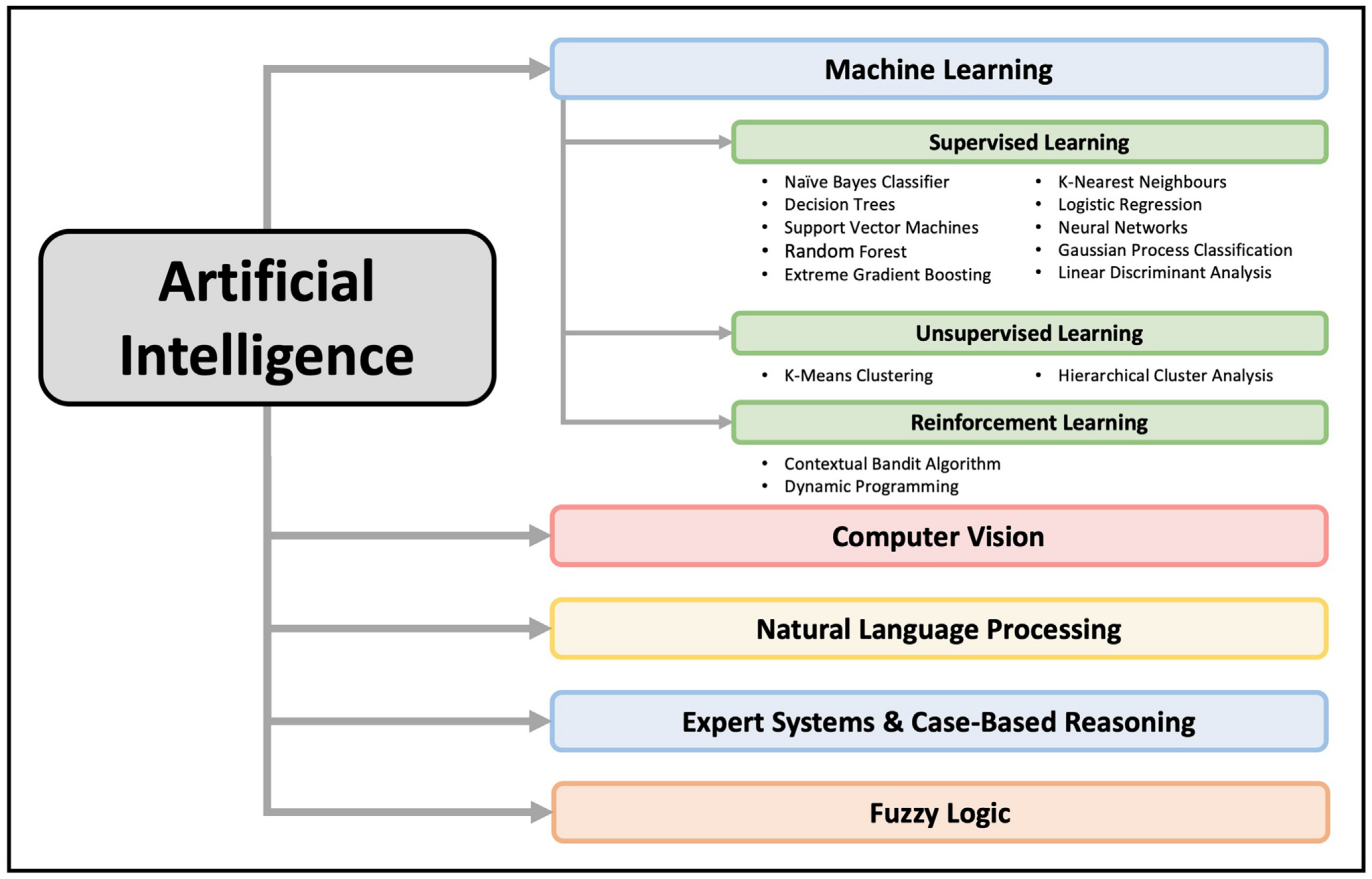
Overview of commonly applied artificial intelligence technologies.

**FIGURE 5:**
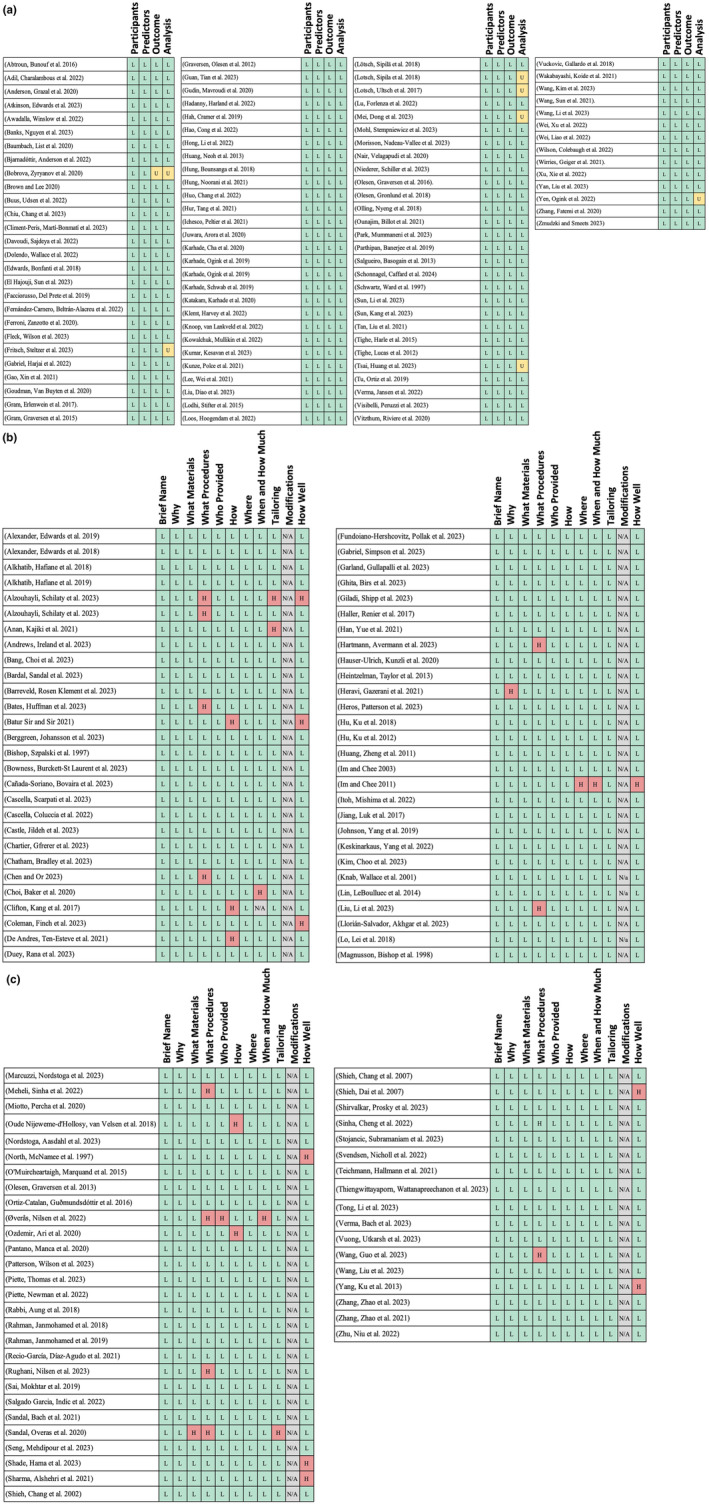
Risk of bias assessment of included studies using PROBAST (Wolff et al., 2019) Checklist for reporting of prediction models (L, low risk of bias; H, high risk of bias; U, Unclear risk of bias) and TIDieR (Hoffmann et al., 2014) Checklist for reporting of interventions (L, low risk of bias; H, high risk of bias; N/A, Not applicable).

**FIGURE 6:**
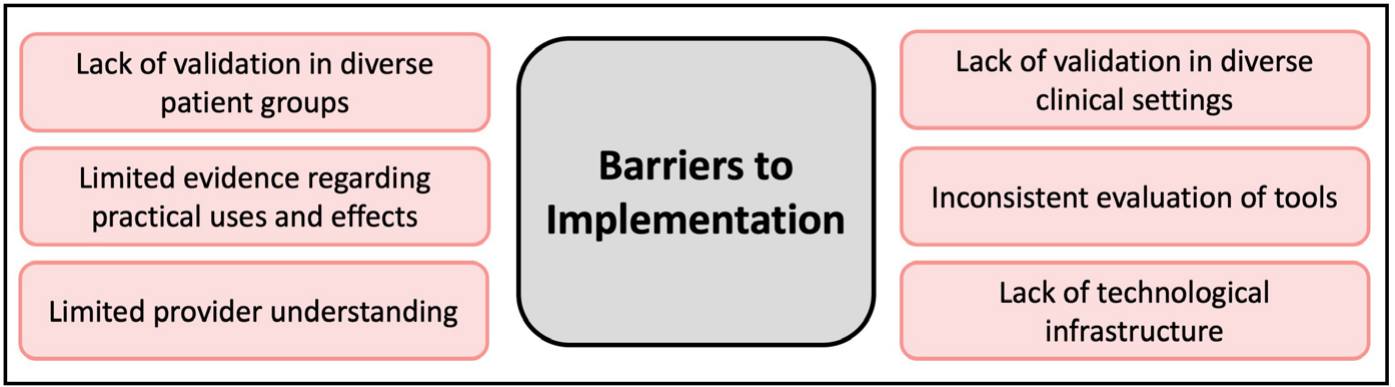
Current barriers to the implementation of AI technologies for pain Management.

The characteristics of the included studies are described in Table S1 and the results of the current literature search are shown in the PRISMA diagram (Figure 1). From the original search which included 17,601 references, 12,435 articles were screened after duplicates were removed, and 551 were selected for full‐text review. This resulted in 197 articles being included in this study. Studies included in this review were published between 1997 and 2023, with a significantly accelerating rate of publication in recent years (Figure 2). Included publications were from 17 different countries, with most from the United States (*n* = 86) and China (*n* = 25). See Figure 3 for an overview of the applications of AI described and Figure 4 for an overview of the types of AI employed. The risk of bias assessment of included studies is summarized in Figure 5. Most included articles were seen to have low risk of bias. However, the main methodological limitation observed across studies was poor reporting regarding the type of AI used, and a lack of rigorous evaluation of the described AI tool. For instance, many studies simply state that AI was used within their intervention, but do not further elaborate regarding the specific branch of AI used (machine learning, NLP, computer vision, etc). Similarly, multiple studies describe an AI tool without evaluating the efficacy of this tool in actual clinical environments. For example, it was commonly noted that studies only evaluate the accuracy of a given tool (i.e. ability to accurately predict analgesic requirements) without studying how this prediction may affect patient outcomes (i.e. does this ultimately translate into improved pain management). Figure 6 describes common barriers to the implementation of such AI technologies for acute and chronic pain management.

### 3.2 Acute pain prediction

The development of AI tools to aid in the management of acute pain is expanding. Using AI to predict patients who are likely to develop acute pain following a surgery or insult may allow clinicians to anticipate appropriate management plans to address this pain. Tools designed for this purpose were described in 22 articles found in the literature (Awadalla et al., 2022; Buus et al., 2022; Davoudi et al., 2022; Dolendo et al., 2022; Gao et al., 2021; Ghita et al., 2023; Guan et al., 2023; Hah et al., 2019; Han et al., 2021; Heravi et al., 2021; Lee, Wei, et al., 2021; Liu, Diao, et al., 2023; Lodhi et al., 2015; Morisson et al., 2023; Olling et al., 2018; O'Muircheartaigh et al., 2015; Sai et al., 2019; Tan et al., 2021; Teichmann et al., 2021; Tighe et al., 2012; Tighe et al., 2015; Zhang et al., 2023). For example Buus et al. described the use of machine learning to identify patients likely to experience high levels of pain following knee arthroplasty to facilitate the initiation of increased analgesia regimens (Buus et al., 2022). Similarly Tan et al. used machine learning to predict parturients at high risk of experiencing breakthrough pain during labour epidural analgesia, allowing for increased personalization of analgesia management (Tan et al., 2021). While methods used to appraise the efficacy of these tools were very heterogeneous in the literature (and were absent in some cases), these applications tended to be accurate in predicting acute pain in patients. However, data regarding how this accuracy translates into patient reported outcomes was limited. This is would be an important area of future work in order to allow the use of these acute pain predictions to ultimately inform the clinical management of pain.

### 3.3 Chronic pain prediction

AI has been used to predict patients likely to transition from acute to chronic pain. This may facilitate the application of analgesia techniques intended to mitigate the risk of pain progression, as well as to plan for management strategies following the potential onset of chronic pain. For exampleSun et al. described the use of machine learning to predict patients likely to develop chronic post‐surgical pain following breast surgery to facilitate subsequent analgesia decisions (such as using regional techniques to potentially reduce the risk of chronic post‐surgical pain) (Sun, Kang, et al., 2023). Furthermore, the use of AI has been used to help clinicians manage patients already experiencing chronic pain, often by predicting the onset of pain exacerbations, such as in patients with sickle cell disease (Vuong et al., 2023).

### 3.4 Medication and dose response prediction

Predicting which patients may respond positively to a certain analgesic medication can improve the ability of clinicians to choose the most effective analgesic agent. This was attempted by Ichesco et al. by using machine learning to predict which patients with fibromyalgia would respond to pregabalin (Ichesco et al., 2021). Similarly Olesen et al. aimed to predict the efficacy of pregabalin in patients with pain due to chronic pancreatitis (Olesen et al., 2013). Furthermore, estimating the most effective dose of a certain analgesic agent according to patient characteristics may allow optimized pain control while minimizing unwanted side effects, and has also been attempted in the literature (Olesen et al., 2018).

### 3.5 Treatment decision‐aids

Deciding which patients may benefit from a given interventional procedure to manage their pain, or a given pharmacological regimen, can be challenging. Using AI to predict a patient's response to a certain therapy may facilitate this decision. For instance, a neural network developed by Kim et al. was able to successfully predict which patients with chronic pain due to foraminal stenosis would benefit from transforaminal steroid injections (Kim et al., 2023). Beyond only predicting response to interventions, the use of AI to explicitly guide the optimal treatment plan for a patient with pain may further alleviate the difficulty of treatment planning. This was attempted by Knab et al. by using an expert system to recommend a treatment regimen for patients with complex chronic pain (including pharmacologic, non‐pharmacologic, and interventional modalities) (Knab et al., 2001). Despite detailed discussion of these interventions in the above studies, little evidence exists as to the impact that these tools may have in actual clinical practice. While the ‘medical appropriateness’ of decisions recommended by the above tools were evaluated by selected experts in multiple studies, no patient care was actually directed by these suggested treatments. As such, further research regarding the real‐world application of these tools is needed.

### 3.6 Opioid‐associated risk prediction

While opioids remain a mainstay of pain management in both the acute and chronic phase, their multiple side effects and potential for dependence (and addiction) require careful attention. Predicting which patients with pain are at risk of long‐term opioid use, and potential opioid misuse, may facilitate the initiation of opioid‐sparing techniques earlier. Similarly, identifying patients treated with opioids that are exhibiting signs of abuse may allow intervention to mitigate problematic behaviour. NLP was used for this purpose by Chatham et al. by using data from electronic health records to identify patients with pain who were experiencing problematic opioid use (Chatham et al., 2023). The use of AI to predict which patients are at high risk of long‐term opioid use following initial prescription has also been extensively described in the literature (Anderson et al., 2020). In particular, the use of machine learning and neural networks to classify patient‐risk based upon patient characteristics, employed treatment modalities and underlying pathology seems to hold particular promise (Gabriel et al., 2022, 2023; Vitzthum et al., 2020; Zhang et al., 2020).

### 3.7 Advanced ultrasound guidance

The use of ultrasound has become routinely incorporated into pain management techniques within the practice of many clinicians. The ability to use ultrasound for real‐time imaging during regional anaesthesia procedures has improved both the safety and efficacy of such approaches (Salinas & Hanson, 2014). The addition of AI to enhance the ability of clinicians to effectively use ultrasound when performing nerve blocks and injections holds the potential to further improve these methods. Potential benefits may include improved regional anaesthesia success rates, decreased injury to surrounding vascular and neural structures, as well as lowered risk of local anaesthetic systematic toxicity. For instance Bowness et al. used machine learning to identify anatomical structures on ultrasound by producing colourful overlays on the generated ultrasound image (Bowness et al., 2023). This not only reportedly improved the rates of block failure but was also judged to reduce the risk of unwanted needle trauma to surrounding anatomical structures (Bowness et al., 2023). Similar to other applications of AI for pain management, the evaluation of the use of AI for advanced ultrasound guidance has not been extensively performed in the clinical environment.

### 3.8 Real‐time treatment tailoring

Following the initiation of a treatment regimen for a patient's pain, the ability to successfully follow and continuously tailor this regimen may allow for improved outcomes. For example, Salgado Garcia et al. described the use of machine learning algorithms in combination with wearable sensors to detect and monitor the self‐administration of opioids after dental surgery (Salgado Garcia et al., 2022). Elsewhere Shieh et al. used fuzzy logic to enhance patient‐controlled analgesia for patients undergoing extracorporeal shock wave lithotripsy by adapting to patients' pattern of medication use (Shieh, Dai, et al., 2007) used case‐based reasoning to adapt physical therapy programs for patients with lower back pain based upon patient characteristics and biomechanical measurements (Recio‐García et al., 2021). Alternatively Coleman et al. used NLP to collect care quality indicators of patients receiving treatment for pain (Coleman et al., 2023). In total, 16 identified articles focused on the use of AI for treatment tailoring (Alzouhayli et al., 2023; Bates et al., 2023; Cañada‐Soriano et al., 2023; Coleman et al., 2023; Fundoiano‐Hershcovitz et al., 2023; Kim et al., 2023; Liu, Li, et al., 2023; North et al., 1997; Ortiz‐Catalan et al., 2016; Recio‐García et al., 2021; Salgado Garcia et al., 2022; Shieh et al., 2002; Shieh, Chang, et al., 2007; Wang, Liu, et al., 2023; Yang et al., 2013).

### 3.9 Self‐management applications

As patients transition from experiencing acute pain to chronic pain, the importance of empowering patients to self‐manage their pain in conjunction with physician oversight has often been emphasized. With the widespread uptake of mobile computing, smartphones and intelligent wearable devices (such as smart watches), the development of electronic applications to facilitate the self‐management of pain have quickly expanded. The incorporation of AI into these tools is now being explored as well. Self‐management tools using AI take many forms, including applications intended to engage patients in cognitive behavioural therapy for chronic pain at home through chatbots (Piette et al., 2022), to suggest tailored at‐home exercise therapy to alleviate chronic musculoskeletal pain (Nordstoga et al., 2023), and to guide appropriate analgesic medication administration following hospital discharge (Piette et al., 2023). Overall, these applications tended to be well‐received by users, who often reported high usability and satisfaction. However, robust data regarding their effect on pain management outcomes is often limited and remains an opportunity for future research.

## 4 DISCUSSION

The great potential of AI to improve pain management is becoming increasingly apparent. Improving provider awareness and understanding of AI, and its potential application to manage pain, is important as we move towards the potential implementation of such tools into clinical practice. To address this, our systematic review aims to provide a basic overview of AI and describe applications incorporating AI for use within pain management. By allowing earlier predictions regarding the pain management needs of our patients, tailoring treatment plans for individual patients, and empowering patients to self‐manage their pain, AI may ultimately improve patient outcomes.

While diverse uses of AI for pain management are appearing at a rapidly growing rate, our experience with the actual implantation of such tools in clinical practice remains sparse. Barriers to the routine clinic implementation of these tools exist, including a lack of validation in diverse patient populations and the relatively small sample sizes used in current studies. Practically speaking, the lack of infrastructure to support these advanced systems, the currently limited provider understanding of these tools and difficult user interfaces that do not easily integrate into already existing healthcare technology setups further hinder the current use of AI for pain management in the clinical domain. Despite promise regarding the efficacy of these tools in carefully designed studies, the evidence regarding the practical implications of these approaches is still largely lacking. The inconsistency among the evaluation of such tools makes appraising the clinical efficacy of these AI applications difficult, especially when considering that the validity of these tools often depends upon the quality of data they are fed. As such, there is a need for new methods to assess newly developed AI tools within the clinical milieu. While it is necessary to study the ability of a given tool to accomplish its intended goal (i.e. ability to predict acute pain in the post‐operative setting), these tools must ultimately be evaluated by their ability to have a clinically meaningful impact (i.e. ability to affect patient pain outcomes). From a practical perspective, the need to implement these systems while upholding ethical patient data sharing standards and adequate data security is essential and will require further work. However, addressing such concerns will be a challenge, especially given the novelty of such technologies. A recent review by Polevikov provides an in‐depth discussion of these issues and outlines current best practices for the implementation of AI in healthcare (Polevikov, 2023).

**FIGURE 7:**
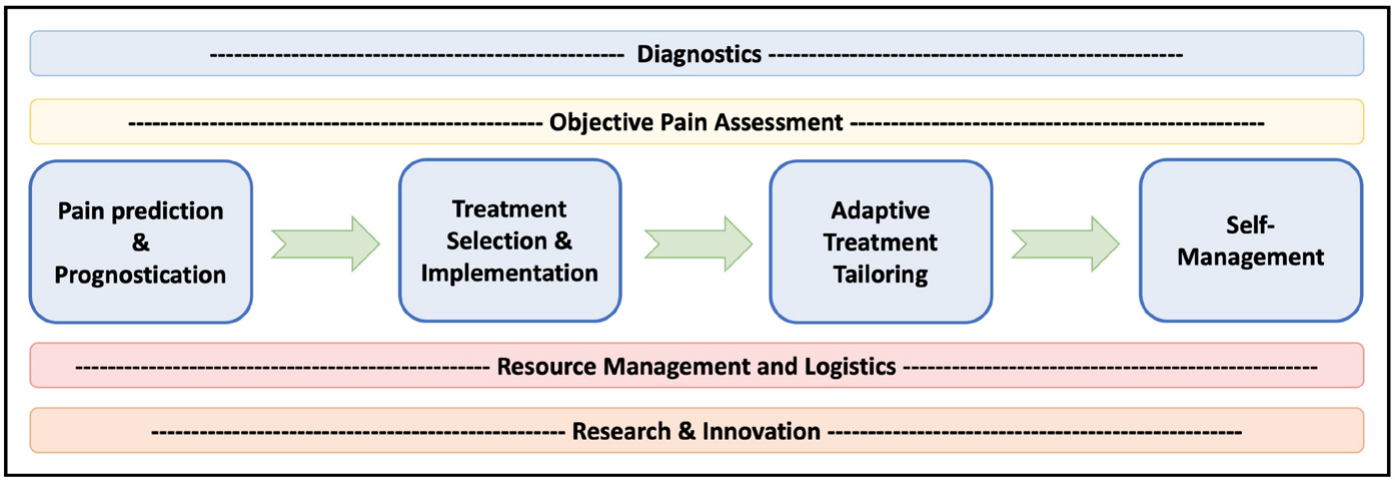
Framework of potential artificial intelligence ecosystem for pain medicine.

Regardless of these barriers, we believe that the future of AI integration into pain medicine extends far beyond interventions merely meant to guide the treatment of pain. Ultimately, treating pain is one step within the patient‐care continuum that may benefit from the use of intelligent computing systems. The true power of AI lies in envisioning a comprehensive system in which AI is used to optimize patient care at every interaction with clinicians. By embracing this wide‐reaching approach, we can truly benefit from the potential of AI to continuously learn, adapt and evolve based upon the vast data collections that can be created throughout the evolution of a patient's care. For instance, the use of AI has already been described in the literature for pain diagnostics (D'Antoni et al., 2022), objective pain assessment (Cascella, Schiavo, et al., 2023), resource management (Bellini et al., 2024) and research initiatives (Lötsch et al., 2022). A brief potential framework incorporating these applications of AI into a patient's care is presented in Figure 7. By combining these distinct applications of AI into a cohesive structure, the integrated intelligent systems have the potential to truly learn from and feed into each stage of a patient's management. For example, allowing the knowledge generated from a diagnostic AI‐based tool to contribute data to an automated pain‐intensity assessment AI‐application holds the potential to improve the latter's accuracy. Similarly, informing tools for prognostication, treatment planning and self‐management from knowledge generated from both diagnostic and assessment tools may further improve the proposed personalized patient management. Ultimately, using the culmination of these tools to populate data collections for research and facilitate patient‐care resource allocation (clinic time management, operating room scheduling, medication and supplies management, etc.) would enable continuous improvement of these very systems. Of course, this level of integration has not yet been attempted in clinical practice and its true feasibility remains a topic of future study. The development of such a system may optimize the efficiency of pain recognition, workup and diagnosis, increasingly tailor treatment plans to individual patients, and contribute to the advancement of scientific discoveries for the treatment of pain. For these systems to reach their full potential though, future efforts needs to be placed upon collecting, storing, cleaning and sharing accurate data collections between groups, institutions and organizations (Dash et al., 2019). This will require strong commitment, raised awareness, funding and widespread infrastructure development to make it a reality.

While our review adheres to previously published methodological frameworks for systematic reviews (Page et al., 2021), this study is not without limitations. For instance, the very large heterogenous collection of literature identified limited our ability to investigate each application of AI in detail, instead focusing on identifying and summarizing trends in the literature. Similarly, the heterogeneity of the described applications limited our ability to compare the accuracy/efficacy of discussed AI tools as many were evaluated using distinct metrics. Finally, the scope of this review was limited due to the English and French language restriction. Future research including a broader range of languages and employing advanced data harmonization techniques may help overcome these limitations.

## 5 CONCLUSION

The integration of AI into pain management carries great potential to improve patient care. As this technology becomes increasingly studied, and eventually used routinely in clinical practice, a basic clinician understanding of such technologies will become increasingly important. Despite the recent advances regarding these intelligent systems, it is important to acknowledge the challenges that continue to impede its incorporation into clinical practice. It is also vital to recognize that the use of AI for pain management is only one facet of a broader landscape, and continued efforts should be directed towards establishing comprehensive systems that seamlessly integrate AI throughout the entire patient care continuum. The future of these systems holds immense potential, although bringing this vision to reality will require continued collaboration from healthcare professionals, researchers and patients alike.

## AUTHOR CONTRIBUTIONS

**RA**: Conceptualization, Data collection, Data Extraction, Formal Analysis, Manuscript Writing. **SW**: Data Collection, Data Extraction, Manuscript Review and Editing. **GG**: Search Strategy Development, Manuscript Review and Editing**. PI**: Conceptualization, Supervision, Manuscript Review and Editing.

## FUNDING INFORMATION

This research did not receive any specific grant from funding agencies in the public, or commercial sectors. The educational and research activities of Dr. Ingelmo are supported by grants of the Montreal Children's Hospital Foundation and of the Louis and Alan Edwards Foundation.

## CONFLICT OF INTEREST STATEMENT

The authors declare that they have no conflict of interest.

## CONSENT

This study does not involve human participants and informed consent was therefore not required.

## Supporting information


Data S1.

Data S2.

Table S1.
