# Supplementary material for: Moving towards the use of artificial intelligence in pain management
Source: Eur J Pain. 2024 Nov 10;29(3):e4748. doi: 10.1002/ejp.4748 (PMC11755729; doi:10.1002/ejp.4748)
Supplement: Supplementary file 1 — Data S1. [file EJP-29-0-s003.pdf]

MEDLINE (Ovid)

4122 records on October 25, 2023

Ovid MEDLINE(R) ALL <1946 to October 24, 2023>

1 pain/ or acute pain/ or chronic pain/ or nociceptive pain/ 180990

2 pain.mp. 882672

3 or/1-2 882672

4 artificial intelligence/ or big data/ or data mining/ or exp *user-computer interface/ or machine learning/ or supervised machine learning/ or unsupervised machine learning/ or natural language processing/ or expert systems/ or fuzzy logic/ or exp neural networks, computer/ or sentiment analysis/ or support vector machine/ 169731

5 ((artificial* or comput* or automat* or ensemble or machine* or multitask* or multi task* or reinforcement or transfer or deep or supervi* or unsupervi* or shallow* or competitive) adj1 (intelligen* or learn* or identifi*)).tw,kf. 184690

6 (computer* adj1 media* adj1 communicat*).tw,kf. 336

7 (natural-language or chat-bot? or chatbot? or (convers* adj2 (agent? or assistant?))).tw,kf. 12092

8 ((bayes* or neural or deep or echo state* or generative adversarial) adj1 (network* or naive* or learning*)).tw,kf. 142469

9 ((automated or comput*) adj1 (heuristic or reasoning or evolutionary or vision)).tw,kf. 9492

10 (data driven or ((data or text) adj1 mining)).tw,kf. 38146

11 (fuzzy adj1 (logic or cluster* or cognit* or inference* or classific* or rule* or system* or control*)).tw,kf. 6143

12 ((adaptive adj (boost* or system*)) or ((ambient or swarm) adj intelligen*) or autoencoder* or auto encod* or backpropagat* or back propagat* or dimensionality reduction or k nearest or learning to rank or metaheuristic* or meta heuristic* or support vector* or ((expert or intelligent* or multiagent* or multi agent*) adj system*) or (markov adj (chain* or process*))).tw,kf. 65738

13 (random* adj2 forest*).tw,kf. 24230

14 ((intelligen* or automat* or comput*) adj2 (recogn* or classific*)).tw,kf. 8817

15 ((case-based or approximate*) adj1 reasoning*).tw,kf. 415

16 ((bio-inspired or evolutionary or genetic or learning or clustering) adj1 algorithm*).tw,kf. 40794

17 or/4-15 394409

18 3 and 17 4122

https://proxy.library.mcgill.ca/login?url=http://ovidsp.ovid.com/ovidweb.cgi?T=JS&NEWS=N&PAGE=main&SHAREDSEARCHID=5eQtF9mxjKJUBXogtqlAciCaRJjFrSUOHuxy0MK77nSviGrHFQSXNTfTLq5wlohDl

Embase (Ovid)

6380 records on October 25, 2023

Embase Classic+Embase <1947 to 2023 October 23>

1 pain/ or chronic pain/ or nociceptive pain/ 469088

2 pain.tw,kf. 1214708

3 or/1-2 1316461

4 artificial intelligence/ or exp *artificial intelligence/ or *big data/ or machine learning/ or exp *machine learning/ or exp *human machine interface/ or *natural language processing/ or *fuzzy logic/ or *computer vision/ or *computer prediction/ 292133

5 ((artificial* or comput* or automat* or ensemble or machine* or multitask* or multi task* or reinforcement or transfer or deep or supervi* or unsupervi* or shallow* or competitive) adj1 (intelligen* or learn* or identifi*)).tw,kf.215466

6 (computer* adj1 media* adj1 communicat*).tw,kf. 365

7 (natural-language or chat-bot? or chatbot? or (convers* adj2 (agent? or assistant?))).tw,kf. 13646

8 ((bayes* or neural or deep or echo state* or generative adversarial) adj1 (network* or naive* or learning*)).tw,kf. 168053

9 ((automated or comput*) adj1 (heuristic or reasoning or evolutionary or vision)).tw,kf. 10246

10 (data driven or ((data or text) adj1 mining)).tw,kf. 45378

11 (fuzzy adj1 (logic or cluster* or cognit* or inference* or classific* or rule* or system* or control*)).tw,kf. 7510

12 ((adaptive adj (boost* or network* or system*)) or ((ambient or swarm) adj intelligen*) or autoencoder* or auto encod* or backpropagat* or back propagat* or dimensionality reduction or k nearest or learning to rank or metaheuristic* or meta heuristic* or support vector* or ((expert or intelligent* or multiagent* or multi agent*) adj system*) or (markov adj (chain* or process*))).tw,kf. 77589

13 (random* adj2 forest*).tw,kf. 29768

14 ((intelligen* or automat* or comput*) adj2 (recogn* or classific*)).tw,kf. 10858

15 ((case-based or approximate*) adj1 reasoning*).tw,kf. 539

16 ((bio-inspired or evolutionary or genetic or learning or clustering) adj1 algorithm*).tw,kf.48554

17 or/4-16 505915

18 3 and 17 6380

https://proxy.library.mcgill.ca/login?url=http://ovidsp.ovid.com/ovidweb.cgi?T=JS&NEWS=N&PAGE=main&SHAREDSEARCHID=7Q5lwqfQCLif31Lf9ruOgVDlzYprUGWGszWIGBjIkv6FCkTUydH70KN9RKmtkWfKG

Web of Science Core Collection (All Editions)

6618 records on October 25, 2023

Web of Science Search Strategy (v0.1)

Database: Web of Science Core Collection

Entitlements:

- WOS.IC: 1993 to 2023

- WOS.CCR: 1985 to 2023

- WOS.SCI: 1900 to 2023

- WOS.AHCI: 1975 to 2023

- WOS.BHCI: 2005 to 2023

- WOS.BSCI: 2005 to 2023

- WOS.ESCI: 2005 to 2023

- WOS.ISTP: 1990 to 2023

- WOS.SSCI: 1900 to 2023

- WOS.ISSHP: 1990 to 2023

Searches:

Search: TS=(pain) AND TS=("big data" OR "data mining" OR "user-computer interface" OR "expert system*" OR "fuzzy logic" OR "sentiment analysis" OR "support vector machine*" OR ((artificial* OR comput* OR automat* OR ensemble OR machine* OR multitask* OR "multi task*" OR reinforcement OR transfer OR deep OR supervi* OR unsupervi* OR shallow* OR competitive) NEAR/1 (intelligen* OR learn* OR identifi*)) OR (computer* NEAR/1 media* NEAR/1 communicat*) OR natural-language OR

chat-bot$ OR chatbot$ OR (convers* NEAR/2 (agent$ OR assistant$)) OR ((bayes* OR neural OR deep OR "echo state*" OR "generative adversarial") NEAR/1 (network* OR naive* OR learning*)) OR ((automated OR comput*) NEAR/1 (heuristic OR reasoning OR evolutionary OR vision)) OR "data driven" OR ((data OR text) NEAR/1 mining) OR (fuzzy NEAR/1 (logic OR cluster* OR cognit* OR inference* OR classific* OR rule* OR system* OR control*)) OR (adaptive NEAR/0 (boost* OR system*)) OR ((ambient OR swarm) NEAR/0 intelligen*) OR autoencoder* OR "auto encod*" OR backpropagat* OR "back propagat*" OR "dimensionality reduction" OR "k nearest" OR "learning to rank" OR metaheuristic* OR "meta heuristic*" OR "support vector*" OR ((expert OR intelligent* OR multiagent* OR "multi agent*") NEAR/0 system*) OR (markov NEAR/0 (chain* OR process*)) OR (random* NEAR/2 forest*) OR ((intelligen* OR automat* OR comput*) NEAR/2 (recogn* OR classific*)) OR ((case-based OR approximate*) NEAR/1 reasoning*) OR ((bio-inspired OR evolutionary OR genetic OR learning OR clustering) NEAR/1 algorithm*))

Date Run: Wed Oct 25 2023 10:51:00 GMT-0400 (Eastern Daylight Time)

Results: 6618

CENTRAL (Cochrane Library)

481 records on October 25, 2023

Search Name: Antel-Pain-AI-FINAL

Date Run: 25/10/2023 12:55:51

Comment:

ID Search Hits

#1 pain:ti,ab,kw 233816

#2 ((artificial*:ti,ab,kw OR comput*:ti,ab,kw OR automat*:ti,ab,kw OR ensemble:ti,ab,kw OR machine*:ti,ab,kw OR multitask*:ti,ab,kw OR ("multi" NEXT task*):ti,ab,kw OR reinforcement:ti,ab,kw OR transfer:ti,ab,kw OR deep:ti,ab,kw OR supervi*:ti,ab,kw OR unsupervi*:ti,ab,kw OR shallow*:ti,ab,kw OR competitive:ti,ab,kw) NEAR/1 (intelligen*:ti,ab,kw OR learn*:ti,ab,kw OR identifi*:ti,ab,kw)) 5216

#3 (computer*:ti,ab,kw NEAR/1 media*:ti,ab,kw NEAR/1 communicat*:ti,ab,kw) 17

#4 (natural-language:ti,ab,kw OR chat-bot?:ti,ab,kw OR chatbot?:ti,ab,kw OR (convers*:ti,ab,kw NEAR/2 (agent?:ti,ab,kw OR assistant?:ti,ab,kw))) 585

#5 ((bayes*:ti,ab,kw OR neural:ti,ab,kw OR deep:ti,ab,kw OR ("echo" NEXT state*):ti,ab,kw OR "generative adversarial":ti,ab,kw) NEAR/1 (network*:ti,ab,kw OR naive*:ti,ab,kw OR learning*:ti,ab,kw)) 2622

#6 ((automated:ti,ab,kw OR comput*:ti,ab,kw) NEAR/1 (heuristic:ti,ab,kw OR reasoning:ti,ab,kw OR evolutionary:ti,ab,kw OR vision:ti,ab,kw)) 165

#7 ("data driven":ti,ab,kw OR ((data:ti,ab,kw OR text:ti,ab,kw) NEAR/1 mining:ti,ab,kw)) 760

#8 (fuzzy:ti,ab,kw NEAR/1 (logic:ti,ab,kw OR cluster*:ti,ab,kw OR cognit*:ti,ab,kw OR inference*:ti,ab,kw OR classific*:ti,ab,kw OR rule*:ti,ab,kw OR system*:ti,ab,kw OR control*:ti,ab,kw)) 92

#9 ((adaptive:ti,ab,kw NEXT (boost*:ti,ab,kw OR system*:ti,ab,kw)) OR ((ambient:ti,ab,kw OR swarm:ti,ab,kw) NEXT intelligen*:ti,ab,kw) OR autoencoder*:ti,ab,kw OR ("auto" NEXT encod*):ti,ab,kw OR backpropagat*:ti,ab,kw OR ("back" NEXT propagat*):ti,ab,kw OR "dimensionality reduction":ti,ab,kw OR "k nearest":ti,ab,kw OR "learning to rank":ti,ab,kw OR metaheuristic*:ti,ab,kw OR ("meta" NEXT heuristic*):ti,ab,kw OR ("support" NEXT vector*):ti,ab,kw OR ((expert:ti,ab,kw OR intelligent*:ti,ab,kw OR multiagent*:ti,ab,kw OR ("multi" NEXT agent*):ti,ab,kw) NEXT system*:ti,ab,kw) OR (markov:ti,ab,kw NEXT (chain*:ti,ab,kw OR process*:ti,ab,kw))) 1969

#10 (random*:ti,ab,kw NEAR/2 forest*:ti,ab,kw) 788

#11 ((intelligen*:ti,ab,kw OR automat*:ti,ab,kw OR comput*:ti,ab,kw) NEAR/2 (recogn*:ti,ab,kw OR classific*:ti,ab,kw)) 490

#12 ((case-based:ti,ab,kw OR approximate*:ti,ab,kw) NEAR/1 reasoning*:ti,ab,kw) 18

#13 ((bio-inspired:ti,ab,kw OR evolutionary:ti,ab,kw OR genetic:ti,ab,kw OR learning:ti,ab,kw OR clustering:ti,ab,kw) NEAR/1 algorithm*:ti,ab,kw)834

#14 {OR #2-#13} 9795

#15 #1 AND #14 in Trials 481
