# Supplementary material for: Moving towards the use of artificial intelligence in pain management
Source: Eur J Pain. 2024 Nov 10;29(3):e4748. doi: 10.1002/ejp.4748 (PMC11755729; doi:10.1002/ejp.4748)
Supplement: Supplementary file 3 — Table S1. [file EJP-29-0-s002.docx]

**Supplemental Table 1. Overview of Included Studies.**

| **Author** | **Year** | **Study Aim** | **Main Type of AI Used** |
| --- | --- | --- | --- |
| (Abtroun, Bunouf et al. 2016) | 2016 | To determine the characteristics of patients who could benefit the most from milnacipran for management of fibromyalgia. | Ward Hierarchical Analysis |
| (Adil, Charalambous et al. 2022) | 2022 | To predict successful reduction or stabilization of opioid usage after spinal cord stimulation using machine learning models. | Logistic Regression, Deep Neural Network |
| (Alexander, Edwards et al. 2019) | 2018 | To evaluate machine learning methods to predict novel, individual patient responses to pregabalin for painful diabetic peripheral neuropathy. | K-Nearest Neighbor, Supervised Fuzzy C-Means |
| (Alexander, Edwards et al. 2018) | 2019 | To predict pregabalin pain reduction outcomes for patients with painful diabetic peripheral neuropathy. | Least Absolute Shrinkage and Selection Operator (LASSO) Regression, K-Nearest Neighbor and Supervised Fuzzy C-Means |
| (Alkhatib, Hafiane et al. 2018) | 2018 | To introduce a new and robust tracking technique of nerve structures in ultrasound images. | Computer Vision |
| (Alkhatib, Hafiane et al. 2019) | 2019 | To explore thirteen most recent deep-learning trackers for nerve tracking and presents a comparative study for the best deep-learning trackers on different types of nerves in ultrasound images. | Convolutional Neural Networks |
| (Alzouhayli, Schilaty et al. 2023) | 2023 | To compare artificial intelligence-guided therapy for low back pain versus supervised in-clinic interventions and supervised home interventions on patient-reported outcomes and strength. | *Not mentioned* |
| (Anan, Kajiki et al. 2021) | 2021 | To evaluate the improvements in musculoskeletal symptoms in workers with neck/shoulder stiffness/pain and low back pain after the use of an exercise-based artificial intelligence (AI)–assisted interactive health promotion system. | Natural Language Processing |
| (Anderson, Grazal et al. 2020) | 2020 | To build a cross-validated model that predicts risk of prolonged opioid use after a specific orthopaedic procedure (ACL reconstruction) and determine its clinical utility. | Random Forest Classifier, Bayesian Belief Network, and Gradient Boosting Machine |
| (Andrews, Ireland et al. 2023) | 2023 | To detail the development and initial pilot testing of a multimodality pain education chatbot. | Natural Language Processing |
| (Atkinson, Edwards et al. 2023) | 2023 | To predict analgesic response to daily oral nonsteroidal anti-inflammatory drugs (NSAIDs) or subcutaneous tanezumab in patients with moderate-to-severe osteoarthritis. | K-Nearest Neighbor |
| (Awadalla, Winslow et al. 2022) | 2022 | To characterize the structure of acute pain trajectories during the postsurgical hospitalization period. | K-Means Clustering |
| (Bang, Choi et al. 2023) | 2023 | To investigate the clinical relevance of deep learning models that predict the onset of cancer pain exacerbation in hospitalized patients. | Recurrent Neural Network, Convolutional Neural Network |
| (Banks, Nguyen et al. 2023) | 2023 | To develop machine-learning predictive models of new opioid use disorder using individual patient characteristics. | Logistic regression, Random Forest Classifier |
| (Bardal, Sandal et al. 2023) | 2023 | To examine if age, gender, or education modify the effectiveness of the SELFBACK intervention (self-management tool). | Case-Based Reasoning |
| (Barreveld, Rosen Klement et al. 2023) | 2023 | To investigate how an artificial intelligence self-management tool affects the daily functions in adults with chronic back and neck pain. | Neural Network |
| (Bates, Huffman et al. 2023) | 2023 | To examine the role of artificial intelligence guided resistance in subjects experiencing lower back pain. | *Not mentioned* |
| (Batur Sir and Sir 2021) | 2021 | To guide physicians in selecting and ranking various alternatives for the treatment of pain in COVID-19 patients using artificial intelligence based decision aids. | Fuzzy logic, Analytic Hierarchy Analysis |
| (Baumbach, List et al. 2020) | 2020 | To predict the individual change in pain intensity following educational and supervised exercise therapy sessions. | Random Forest Classifier |
| (Berggreen, Johansson et al. 2023) | 2023 | To develop a deep learning semantic segmentation model to identify the femoral nerve in ultrasound images. | Convolutional Neural Network |
| (Bishop, Szpalski et al. 1997) | 1997 | To develop a neural network analysis system to analyze specific characteristics of trunk motion (shape, velocity, and symmetry of movements) to prescribe and follow up specific rehabilitation. | Neural Network |
| (Bjarnadóttir, Anderson et al. 2022) | 2022 | To investigate the performance of machine learning algorithms for predicting the risk of chronic opioid therapy. | Logistic Regression, Least Absolute Shrinkage and Selection Operator (LASSO) Regression |
| (Bobrova, Zyryanov et al. 2020) | 2020 | To develop a calculator for personalized risk assessment of opioid-associated drug resistance in patients with pancreas cancer. | K-Nearest Neighbor, Random Forest Classifier, Gradient Boosting, Decision Trees, Neural Network, Linear Support Vector Machine |
| (Bowness, Burckett-St Laurent et al. 2023) | 2023 | To evaluate the accuracy of the artificial-intelligence colour overlay on ultrasound and its perceived influence on risk of adverse events or block failure. | Convolutional Neural Network |
| (Brown and Lee 2020) | 2020 | To predict the probability of developing or maintaining moderate-to-Severe chronic pain 7–10 years into the future. | Least Absolute Shrinkage and Selection Operator (LASSO) Regression |
| (Buus, Udsen et al. 2022) | 2022 | To use traditional statistics and machine learning to develop prediction models that identify patients likely to have increased care needs related to managing function and pain following total knee arthroplasty. | K-Nearest Neighbor, Support Vector Machine, Recursive Partitioning and Regression Tree (CART) Analysis, Random Forest Classifier, Logistic Regression |
| (Cañada-Soriano, Bovaira et al. 2023) | 2023 | To assess the performance of different machine learning algorithms to classify lumbar sympathetic blocks carried out in patients diagnosed with lower limbs Complex Regional Pain Syndrome as successful or failed. | Natural Language Processing |
| (Cascella, Scarpati et al. 2023) | 2023 | To investigate the capabilities of a specific deep learning model and explore its potential for enhancing the telemedicine approach to cancer pain management. | Artificial Neural Network, K-Nearest Neighbours, Random Forest Classifier, Support Vector Machine |
| (Cascella, Coluccia et al. 2022) | 2022 | To identify patients with cancer pain who require increased attention and calibrated follow-up programs. | Least Absolute Shrinkage and Selection Operator (LASSO) Regression, Random Forest Classifier, Gradient Boosting Machine, Artificial Neural Network |
| (Castle, Jildeh et al. 2023) | 2023 | To develop a predictive machine learning model to identify prognostic factors for continued opioid prescriptions after arthroscopic meniscus surgery. | Naïve Bayes Classifier |
| (Chartier, Gfrerer et al. 2023) | 2023 | To develop a machine learning framework capable of automatically interpreting pain drawings to predict surgical outcomes. This platform will allow surgeons with less clinical experience, neurologists, primary care practitioners, and even patients to better understand candidacy for headache surgery. | Random Forest Classifier |
| (Chatham, Bradley et al. 2023) | 2023 | To determine whether regular text-based expressions, a highly interpretable natural language processing technique, could automate a validated clinical tool to expedite the identification of problematic opioid use in the electronic health record. | Natural Language Processing |
| (Chen and Or 2023) | 2023 | To design of a machine learning-based system for lower-limb exercise training for patients with knee pain that features three main components: video demonstration of exercises, real-time movement feedback, and tracking of exercise progress. | *Not mentioned* |
| (Chiu, Chang et al. 2023) | 2023 | To develop machine learning based radiomic models based on pre-treatment imaging for predicting the outcomes of lumbar nucleoplasty for lumbar degenerative disk disease. | Support Vector Machine, Gradient Boosting Machine, Extreme Gradient Boosting, Random Forest Classifier |
| (Choi, Baker et al. 2020) | 2020 | To demonstrate the steps required to develop a prototype cancer pain assessment mobile app based on algorithms derived from a science-based clinical guideline. | Expert System |
| (Clifton, Kang et al. 2017) | 2017 | To develop a new hybrid model for the dynamics of subjective pain that consists of a dynamical systems approach using differential equations to predict future pain levels, as well as a statistical approach tying system parameters to patient data (both personal characteristics and medication response history). | Least Absolute Shrinkage and Selection Operator (LASSO) Regression |
| (Climent-Peris, Martí-Bonmatí et al. 2023) | 2023 | To determine whether magnetic resonance imaging (MRI) texture analysis could predict the prognosis of patients with non-specific chronic low back pain. | Random Forest Classifier |
| (Coleman, Finch et al. 2023) | 2023 | To identify pain care quality indicators and assess patterns across different clinic visit types using natural language processing chiropractic clinic documentation. | Natural Language Processing |
| (Davoudi, Sajdeya et al. 2022) | 2023 | To evaluate prediction bias in machine learning models used for predicting acute postoperative pain. | Decision Trees |
| (De Andres, Ten-Esteve et al. 2021) | 2021 | To investigate the usefulness of imaging biomarkers, functional connectivity and volumetry of the whole brain in patients with failed back surgery syndrome and to create a clinical patient-based decision support system combining neuroimaging and clinical data for predicting the effectiveness of neurostimulation therapy after a trial phase. | Logistic Regression, Decision Trees, Linear Discriminant Analysis, Gaussian Naive Bayes, K-neighbors, Support Vector Machine |
| (Dolendo, Wallace et al. 2022) | 2022 | To identify risk factors and develop machine-learning-based models to predict patients who are at higher risk for postoperative opioid use after mastectomy. | Logistic Regression, Ridge Regression; Least Absolute Shrinkage and Selection Operator (LASSO) Regression, Elastic Net Regression |
| (Duey, Rana et al. 2023) | 2023 | To predict daily pain levels using speech recordings from personal smartphones of a cohort of patients with diagnosed neurological spine disease. | K-Nearest Neighbor |
| (Edwards, Bonfanti et al. 2018) | 2018 | To predict outcomes for pregabalin-treated painful diabetic peripheral neuropathy patients based on 4 weeks of pain and pain-related sleep interference data. | K-Nearest Neighbor |
| (El Hajouji, Sun et al. 2023) | 2023 | To develop a machine-learning algorithm to predict the risk of OR-AE following surgery using Medicaid data with external validation across states. | Logistic Regression, Ridge Regression, Least Absolute Shrinkage and Selection Operator (LASSO) Regression, Elastic Net Regression, Random Forest Classifier, Extreme Gradient Boosted Trees, Deep Neural Network |
| (Facciorusso, Del Prete et al. 2019) | 2019 | To assess the efficacy of repeat celiac plexus neurolysis and to build an artificial neural network model able to predict pain response. | Artificial Neural Network |
| (Fernández-Carnero, Beltrán-Alacreu et al. 2022) | 2022 | To develop a predictive learning approach to determine prognostic value for musculoskeletal mobilization techniques and to identify the most important predictive factors for recovery in chronic neck pain subjects in four key areas: the number of treatments, time of treatment, reduction of pain, and range of motion increase. | Logistic Regression, Support Vector Machine, Kernel, Decision Trees, Random Forest Classifier, Neural Networks |
| (Ferroni, Zanzotto et al. 2020). | 2020 | To analyze the performance of a customized machine learning based decision support system that generates automatic predictors for medication overuse in migraine patients. | Support Vector Machine, Random Optimization |
| (Fleck, Wilson et al. 2023) | 2023 | To examine the relative influence of various cognitive functional domains on engagement with an online pain self-management program. | Fuzzy Logic |
| (Fritsch, Steltzer et al. 2023) | 2023 | To use machine learning to classify the effectiveness of analgesic medication cocktails for patients’ pain. | Artificial Neural Network |
| (Fundoiano-Hershcovitz, Pollak et al. 2023) | 2023 | To present an analytical framework for personalized pain management. | Decision Trees |
| (Gabriel, Harjai et al. 2022) | 2022 | To develop machine learning algorithms incorporating pain and opioid features to predict the need for outpatient opioid refills following ambulatory surgery. | Neural Network, Logistic Regression, Random Forest Classifier, Support Vector Machine |
| (Gabriel, Simpson et al. 2023) | 2023 | To develop predictive models for persistent opioid use following lower extremity joint arthroplasty and determine if ensemble learning and an oversampling technique may improve model performance. | Logistic Regression, Random Forest Classifier, Neural Network, Random Forest Classifier, Balanced Bagging Classifier, Support Vector Classifier |
| (Gao, Xin et al. 2021) | 2021 | To evaluate the accuracy of an artificial neural network model for predicting postoperative pain following root canal treatment. | Neural Network |
| (Garland, Gullapalli et al. 2023) | 2023 | To predict if a mindfulness session would decrease pain and stress ratings in Opioid-treated chronic pain patients  using biometric data (e.g., heart rate, HRV, pulse oximeter, accelerometer, calorie, and step count) captured from a wristwatch. | Logistic Regression, Decision Tree, Random Forest Classifier |
| (Ghita, Birs et al. 2023) | 2023 | To develop a deep learning model to predict personalized nociception requirements in the post-anesthesia care unit. | Convolutional Neural Network |
| (Giladi, Shipp et al. 2023) | 2023 | To develop a machine learning approach leveraging preoperative patient-reported date and electronic health record data to predict persistent opioid use after upper extremity surgery. | Logistic Regression, Random Forest Classifier |
| (Goudman, Van Buyten et al. 2020) | 2020 | To predict responders of high frequency spinal cord stimulation for patients with failed back surgery syndrome. | Logistic Regression, Linear Discriminant Analysis, Classification and Regression Trees, Random Forest Classifier |
| (Gram, Erlenwein et al. 2017). | 2017 | To investigate if electroencephalography (EEG) during rest or pain before treatment could predict the analgesic response to opioid for patients undergoing total hip replacement. | Support Vector Machine |
| (Gram, Graversen et al. 2015) | 2015 | To predict the analgesic response to opioids based on the pre-operative electroencephalography (EEG) on an individualized basis in patients undergoing hip replacement surgery. | Support Vector Machine |
| (Graversen, Olesen et al. 2012) | 2012 | To identify electroencephalographic (EEG) biomarkers for the analgesic effect of pregabalin in patients with chronic visceral pain. | Support Vector Machine |
| (Guan, Tian et al. 2023) | 2023 | To develop an early warning model for predicting pain after transcatheter arterial chemoembolization to enable the implementation of preventive analgesic measures. | Random Forest Classifier, Support Vector Machine, Artificial Neural Network, Naive Bayes Model, Decision Tree |
| (Gudin, Mavroudi et al. 2020) | 2020 | To explore the potential of machine learning methods to discriminate chronic pain patients into ones who will benefit from non-opioid treatments and ones who will not, aiming to personalize their treatment. | Support Vector Machine |
| (Hadanny, Harland et al. 2022) | 2022 | To develop machine learning based predictive models of long-term spinal cord stimulation response. | K-Means Clustering, Random Forest Classifier |
| (Hah, Cramer et al. 2019) | 2019 | To identify patient clusters to predict remote postoperative pain resolution, opioid cessation, and full recovery in patients undergoing surgery. | K-Means Clustering |
| (Haller, Renier et al. 2017) | 2017 | To evaluate natural language processing for risk assessment of patients considered for opioid therapy as a means of predicting opioid abuse. | Natural Language Processing |
| (Hao, Cong et al. 2022) | 2022 | To investigate use of multidata analysis based on an artificial neural network to predict long-term pain outcomes after microvascular decompression in patients with trigeminal neuralgia and to explore key predictors. | Artificial Neural Network |
| (Hartmann, Avermann et al. 2023) | 2023 | To investigate the extent to which artificial intelligence-assisted exercise recommendations can reduce pain and pain‐related impairments in daily life for patients with low back pain. | *Not mentioned* |
| (Hauser-Ulrich, Kunzli et al. 2020) | 2020 | To describe the design and implementation of a chatbot for the self-management of pain and to present findings from a pilot randomized controlled trial, in which effectiveness, influence of intention to change behavior, pain duration, working alliance, acceptance, and adherence were evaluated. | Natural Language Processing |
| (Heintzelman, Taylor et al. 2013) | 2013 | To test the feasibility of using text mining to depict meaningfully the experience of pain in patients with metastatic prostate cancer, to identify novel pain phenotypes, and to propose methods for longitudinal visualization of pain status. | Natural Language Processing |
| (Heravi, Gazerani et al. 2021) | 2021 | To estimate pain after coronary angiography based on vital signs for determining best patient position by using artificial neural networks | Artificial Neural Network |
| (Heros, Patterson et al. 2023) | 2023 | To compare the utilization of the common subjective patient-reported outcomes with objective measures captured through a wearable device for predicting the response to neurostimulation therapy using machine learning. | Random Forest Classifier |
| (Hong, Li et al. 2022) | 2022 | To investigate clinical variables and inflammatory biomarkers as risk factors for chronic postsurgical pain after hepatectomy to build a reliable machine learning model to predict the occurrence of chronic postsurgical pain. | Support Vector Machines, Random Forest Classifier |
| (Hu, Ku et al. 2018) | 2012 | To use machine learning and data mining techniques to predict analgesic requirements and patient-controlled analgesia (PCA) readjustments in patients admitted to hospital with acute pain. | Decision Tree, K-Nearest Neighbor |
| (Hu, Ku et al. 2012) | 2018 | To develop a computational approach to predict analgesic consumption using data from patient-controlled analgesia demand behavior over time. | K-Means Clustering, Multimodal Regression Tree |
| (Huang, Neoh et al. 2013) | 2011 | To validate the use of artificial neural network models for predicting myofascial pain control after dry needling and to compare the predictive capability of artificial neural networks with that of support vector machine and multiple linear regression. | Support Vector Machine, Multiple Linear Regression, Artificial Neural Network |
| (Huang, Zheng et al. 2011) | 2013 | To analyze self-reported data collected from biopsychosocial-based treatments for chronic pain, in order to develop a personalized self-management system for chronic pain. | Support Vector Machine, Decision  Trees |
| (Hung, Bounsanga et al. 2018) | 2018 | To identify predictors of pain associated with arthritis to allow the development of cost-effective individualized care management programs. | Decision Trees |
| (Hung, Noorani et al. 2021) | 2021 | To determine if pre-treatment regional brain morphology-based machine learning models can prognosticate 1-year response to gamma knife radiosurgery for trigeminal neuralgia. | Support Vector Machine |
| (Huo, Chang et al. 2022) | 2022 | To explore the value of data mining model in evaluating the treatment effect of acupuncture on patients with cervical spondylosis and neck pain. | Artificial Neural Network |
| (Hur, Tang et al. 2021) | 2021 | To utilize preoperative insurance claims data to predict postoperative opioid refill and new persistent opioid use in opioid-naive patients in patients undergoing surgery. | Support Vector Machine |
| (Ichesco, Peltier et al. 2021) | 2021 | To predict whether a patient would respond differentially to either milnacipran or pregabalin for the treatment of fibromyalgia. | Support Vector Machine |
| (Im and Chee 2003) | 2003 | To develop a decision support system using fuzzy logic that would support nurses' decisions about cancer pain management, especially for ethnic minority cancer patients. | Fuzzy Logic, Expert System |
| (Im and Chee 2011) | 2011 | To develop an initial version of computer software that could assist nurses’ decision making about cancer pain reported by women from diverse cultural groups. | Fuzzy Logic, Expert System |
| (Itoh, Mishima et al. 2022) | 2022 | This study aimed to explore the effects of web-based video patient education and strengthening exercise therapy, using a mobile messaging app, on work productivity and pain in patients with chronic low back pain receiving pharmacological treatment. | Natural Language Processing |
| (Jiang, Luk et al. 2017) | 2017 | To investigate the feasibility and applicability of a support vector machine algorithm in classifying patients with low back pain who would obtain satisfactory or unsatisfactory progress after a functional restoration rehabilitation program. | Support Vector Machine |
| (Johnson, Yang et al. 2019) | 2019 | To demonstrate the feasibility of using objective, physiologic measurements obtained from a wearable device during an acute pain crisis to predict patient-reported pain scores using machine learning techniques to individualize treatment. | Ridge Regression, Least Absolute Shrinkage and Selection Operator (LASSO) Regression, Support Vector Machine |
| (Juwara, Arora et al. 2020) | 2020 | To identify specific pain characteristics that can help determine which patients may be susceptible to neuropathic after breast cancer surgery and assess the utility of machine learning models for prediction. | Least square, Ridge Regression, Elastic Net Regression, Random Forest Classifier, Neural Network |
| (Karhade, Cha et al. 2020) | 2019 | To develop algorithms to predict prolonged opioid prescription after surgery for lumbar disc herniation. | Random Forest Classifier, Stochastic Gradient Boosting, Neural Network, Support Vector Machine, Elastic-Net Regression |
| (Karhade, Ogink et al. 2019) | 2019 | To develop machine learning algorithms for preoperative prediction of prolonged opioid prescriptions after total hip arthroplasty. | Random Forest Classifier, Stochastic Gradient Boosting, Neural Network, Support Vector Machine, Elastic-Net Regression |
| (Karhade, Ogink et al. 2019) | 2019 | To develop machine learning algorithms for preoperative prediction of sustained opioid prescription after anterior cervical discectomy and fusion. | Random Forest Classifier, Stochastic Gradient Boosting, Neural Network, Support Vector Machine, Elastic-Net Regression |
| (Karhade, Schwab et al. 2019) | 2020 | To develop supervised machine learning algorithms for preoperative prediction of prolonged opioid prescription use in opioid-naive patients following lumbar spine surgery. | Random Forest Classifier, Stochastic Gradient Boosting, Neural Network, Support Vector Machine, Elastic-Net Regression |
| (Katakam, Karhade et al. 2020) | 2020 | To develop machine learning algorithms for preoperative prediction of prolonged opioid prescriptions after total knee arthroplasty. | Random Forest Classifier, Stochastic Gradient Boosting, Neural Network, Support Vector Machine, Elastic-Net Regression |
| (Keskinarkaus, Yang et al. 2022) | 2022 | To use hospital measurements, questionnaires and the implementation of a home biosensor measurement device to evaluate pain in patients with chronic low back pain to help in treatment personalization. | Support Vector Machine |
| (Kim, Choo et al. 2023) | 2023 | To develop deep-learning algorithms that can automatically prescribe orthotic insoles to patients with foot pain and assess the algorithms accuracy. | Deep Neural Network |
| (Klemt, Harvey et al. 2022) | 2022 | To accurately predict extended opioid use following primary total knee arthroplasty. | Stochastic Gradient Boosting, Artificial Neural Network, Random Forest Classifier, K-Nearest Neighbor, and Elastic-Net Regression |
| (Knab, Wallace et al. 2001) | 2001 | To test whether computer-based decision support could enhance the ability of primary care physicians to manage chronic pain. | Expert System |
| (Knoop, van Lankveld et al. 2022) | 2022 | To develop and internally validate a machine learning model to predict non-recovery in acute low back pain. | Extreme Gradient Boosting |
| (Kowalchuk, Mullikin et al. 2022) | 2022 | To identify a subset of patients with the highest rate of pain flare after spine Stereotactic body radiotherapy to optimize prophylactic medication administration. | Decision Trees |
| (Kumar, Kesavan et al. 2023) | 2023 | To develop and cross-validate robust predictive models for postoperative fentanyl analgesic requirement and other related outcomes in patients undergoing major breast surgery. | Support Vector Machine, Random Forest Classifier, Neural Network |
| (Kunze, Polce et al. 2021) | 2021 | To develop machine learning algorithms to predict prolonged opioid use after hip arthroscopy in opioid-naïve patients. | Random Forest Classifier, Stochastic Gradient Boosting, Neural Network, Support Vector Machine, Elastic-Net Regression |
| (Lee, Wei et al. 2021) | 2021 | To predict individualized opioid requirements for patients after total joint replacement. | K-Nearest Neighbor, Logistic Regression, Support Vector Machine, Neural Network, Random Forest Classifier, Extreme Gradient Boosting |
| (Lin, LeBoulluec et al. 2014) | 2014 | To develop a decision-making framework for adaptive pain management based upon a decision support system (DSS) that simultaneously minimizes treatment medication dosages and optimizes patient pain outcomes. | Dynamic Programming, Neural Network |
| (Liu, Diao et al. 2023) | 2023 | To evaluate the effect of artificial intelligent patient-controlled intravenous analgesia in older patients after laparoscopic radical resection of colorectal cancer. | *Not mentioned* |
| (Liu, Li et al. 2023) | 2007 | To construct and validate a model for the preoperative evaluation of patients’ risk for postoperative pain after tonsillectomy. | Linear Discriminant Analysis |
| (Llorián-Salvador, Akhgar et al. 2023) | 2023 | To assess the effectiveness of machine learning models trained on radiomics, semantic and clinical features to estimate pain response of patients with painful spinal bone metastases undergoing palliative radiation therapy. | Random Forest Classifier, Support Vector Machine |
| (Lo, Lei et al. 2018) | 2023 | To evaluate the feasibility of an artificial intelligence-embedded mobile app to assist people with chronic neck and back pain to self-manage their condition. | Artificial Neural Network |
| (Lodhi, Stifter et al. 2015) | 2015 | To build predictive models for determining the factors that influence pain in end-of-life patients using information from the electronic health record. | Decision Trees, K-Nearest Neighbors, Support Vector Machine, Logistic Regression |
| (Loos, Hoogendam et al. 2022) | 2022 | To develop and validate prediction models for clinically important improvement in pain and hand function 12 months after surgery for thumb carpometacarpal osteoarthritis. | Logistic Regression, Random Forest Classifier, Gradient Boosting Machine |
| (Lotsch, Sipila et al. 2018) | 2018 | To create a predictive tool for persistent pain, or its absence, following breast cancer surgery and adjuvant therapies. | Bayesian Decision Theory, Kullback-Leibler Divergence |
| (Lotsch, Ultsch et al. 2017) | 2018 | To test how accurately a patients' performance in a preoperatively performed tonic cold pain test could predict persistent post-surgery pain. | K-Nearest Neighbor |
| (Lötsch, Sipilä et al. 2018) | 2017 | To use supervised machine learning to predict pain persistence for women undergoing breast cancer surgery. | Random Forest Classifier |
| (Lu, Forlenza et al. 2022) | 2022 | To develop and validate a machine-learning algorithm that can reliably and effectively predict prolonged opioid consumption in patients following elective knee arthroscopy. | Support Vector Machine, Random Forest Classifier, Extreme Gradient Boosting |
| (Magnusson, Bishop et al. 1998) | 1998 | To determine specific characteristics of trunk motion associated with long-term dysfunction caused by low back pain of various origin, to determine if a neural network analysis system can be effective in distinguishing between patterns, and to determine if the rehabilitation has an effect on range and pattern of motion. | Neural Network |
| (Marcuzzi, Nordstoga et al. 2023) | 2023 | To determine the effect of individually tailored self-management support delivered via an artificial intelligence–based app for musculoskeletal health. | Case-Based Reasoning |
| (Meheli, Sinha et al. 2022) | 2022 | To evaluate the perceived needs, engagement, and effectiveness of an artificial intelligence conversational agent with regard to mental health outcomes among real-world users who reported chronic pain. | *Not mentioned* |
| (Mei, Dong et al. 2023) | 2023 | To develop an artificial intelligence model to analyze patient preferences for treatment of musculoskeletal pain to ultimately improve clinical management. | Extreme Gradient Boosting |
| (Miotto, Percha et al. 2020) | 2020 | To evaluate the feasibility of automatically distinguishing acute low back pain episodes by analyzing free-text clinical notes. | Convolutional Neural Network |
| (Mohl, Stempniewicz et al. 2023) | 2023 | To estimate the risk of a patient with osteoarthritis developing chronic opioid use within one year of a new opioid prescription by using electronic health record data and predictive models. | Logistic regression, Elastic Net Regression, Support Vector Machine, Random Forest Classifier |
| (Morisson, Nadeau-Vallee et al. 2023) | 2023 | To evaluate the ability of a machine-learning algorithm to predict moderate to severe acute postoperative pain based on intraoperative nociception level index values. | Support Vector Machine, Random Forest Classifier, Extreme Gradient Boosting, Elastic Net Regression, Logistic Regression |
| (Nair, Velagapudi et al. 2020) | 2020 | To develop machine learning models to predict postoperative opioid requirements in patients undergoing ambulatory surgery. | Logistic Regression, Naive-Bayes Classifier, Neural Network, Random Forest Classifier, Extreme Gradient Boosting |
| (Niederer, Schiller et al. 2023) | 2023 | To develop a multivariable prediction model to determine factors of rehabilitation success and the risk for a future healthcare use in patients with high-grade, chronic low back pain. | Random Forest Classifier, Decision Trees |
| (Oude Nijeweme-d'Hollosy, van Velsen et al. 2018) | 2017 | To develop a clinical decision support system to support patients with LBP in their self-referral to primary care. | Decision Tree, Random Forest Classifier |
| (Nordstoga, Aasdahl et al. 2023) | 2023 | To explore if the baseline duration and intensity of low back pain influence the effectiveness of an artificial intelligence-based self-management application that supports tailored and evidence-based self-management of nonspecific low back pain. | Case Based Reasoning |
| (North, McNamee et al. 1997) | 1997 | To develop an artificial neural network to optimize the position of implanted spinal cord stimulators in patients with chronic pain. | Artificial Neural Network |
| (O'Muircheartaigh, Marquand et al. 2015) | 2015 | To develop a post-surgical pain prediction model using regional cerebral blood flow indices acquired prior to and following surgical intervention | Gaussian Process Classification |
| (Olesen, Graversen et al. 2016) | 2018 | To investigate if timing of medical treatment is associated with the analgesic effect of pregabalin or placebo in patients with chronic pancreatitis | Logistic Regression, Support Vector Machine |
| (Olesen, Gronlund et al. 2018) | 2013 | To investigate whether data processing with support vector machine learning could predict required opioid dose in cancer pain patients. | Support Vector Machine |
| (Olesen, Graversen et al. 2013) | 2016 | To evaluate the ability of quantitative sensory testing to predict the analgesic effect of pregabalin and placebo in patients with chronic pancreatitis. | Support Vector Machine |
| (Olling, Nyeng et al. 2018) | 2018 | To generate prediction models for odynophagia needing prescription pain medication during external beam lung radiotherapy for non-small cell and small-cell lung cancer. | Least Absolute Shrinkage and Selection Operator (LASSO) Regression, Elastic Net Regression, Support Vector Machine |
| (Ortiz-Catalan, Guðmundsdóttir et al. 2016) | 2016 | To determine whether re-engagement of central and peripheral circuitry involved in motor execution could reduce phantom limb pain via competitive plasticity and reversal of cortical reorganisation. | Artificial Neural Network |
| (Ounajim, Billot et al. 2021) | 2021 | To develop machine learning models that could predict long-term spinal cord stimulation success in individual patients. | Naive Bayes Classifier, Artificial Neural Networks, Support Vector Machine, Classification and Regression Trees, Random Forest Classifier |
| (Øverås, Nilsen et al. 2022) | 2022 | To explore if multimorbidity (≥ 2 long-term conditions) influences the effect of an artificial intelligence-based application delivering evidence-based tailored self-management support to people with low back pain. | Case Based Reasoning |
| (Ozdemir, Ari et al. 2020) | 2020 | To identify, in the early stages, patients' diabetic neuropathy and pain by using the artificial neural network-based computerized clinical decision support systems. | Artificial Neural Network |
| (Pantano, Manca et al. 2020) | 2020 | To explore whether an unsupervised learning algorithm can identify patients at high risk of breakthrough cancer pain. | Hierarchical Clustering |
| (Park, Mummaneni et al. 2023) | 2023 | To evaluate the performance of different supervised machine learning algorithms to predict achievement of minimum clinically important difference in neck pain after surgery in patients with cervical spondylotic myelopathy. | Logistic Regression, Support Vector Machine, Decision Tree, Random Forest Classifier, K–Nearest Neighbors, Extreme Gradient Boosting |
| (Parthipan, Banerjee et al. 2019) | 2019 | To use a machine learning approach to identify patients prescribed a combination of selective serotonin reuptake inhibitors and prodrug opioids postoperatively and to examine the effect of this combination on postoperative pain control. | Natural Language Processing (NLP), Elastic Net Regression, Support Vector Machine, Random Forest Classifiers |
| (Patterson, Wilson et al. 2023) | 2023 | To assess the feasibility of using digital biomarkers collected from wearables during Spinal Cord Stimulation treatment to predict pain and patient-reported outcomes in chronic pain patients. | Logistic Regression, Support Vector Machine, K-Nearest Neighbors, Random Forest Classifiers, Decision Trees |
| (Piette, Thomas et al. 2023) | 2022 | To evaluate whether an intervention based on reinforcement learning can personalize interactions with patients with pain discharged from the emergency department. | Contextual Bandit Algorithm |
| (Piette, Newman et al. 2022) | 2023 | To determine if a cognitive behavioral therapy for chronic pain program that personalizes patient treatment using reinforcement learning, is noninferior to standard telephone cognitive behavioral therapy for chronic pain. | Contextual Bandit Algorithm |
| (Han, Yue et al. 2021) | 2021 | To predict acute postoperative pain based on pre-surgery physiological measures to provide valuable insights into individualized, effective analgesic strategies, thus helping improve the analgesic efficacy. | Linear Discriminant Analysis |
| (Rabbi, Aung et al. 2018) | 2018 | To report the preliminary efficacy of a mobile phone app that uses machine learning on sensor-based and self-reported physical activity data to find routine behaviors and automatically generate physical activity recommendations that are similar to existing behavior for individuals with chronic back pain. | BIRCH Clustering Algorithm |
| (Rahman, Janmohamed et al. 2018) | 2018 | To use data mining and machine learning methods to define a new measure of pain volatility and predict future pain volatility levels from users of a pain management app based upon demographic, clinical, and app use features. | K-Means Clustering, Logistic Regression, Least Absolute Shrinkage and Selection Operator (LASSO) Regression, Random Forests Classifiers, Support Vector Machines |
| (Rahman, Janmohamed et al. 2019) | 2019 | To increase the interpretability of previously developed pain volatility models by identifying the most important features that distinguish high from low volatility users; and (consolidate prediction results from models derived from multiple random subsamples while addressing the class imbalance issue. | Logistic regression, Least Absolute Shrinkage and Selection Operator (LASSO) Regression, Random Forest Classifier |
| (Recio-García, Díaz-Agudo et al. 2021) | 2021 | To describe and evaluate a machine-learning based configuration software associated to a therapy machine that executes individualized back segment mobilisations. | Case Based Reasoning |
| (Rughani, Nilsen et al. 2023) | 2023 | To explore a tailored self-management support system for low back pain via an artificial intelligence smartphone app. | *Not mentioned* |
| (Sai, Mokhtar et al. 2019) | 2019 | To assess the utility of electroencephalography as an objective marker of pain during the first stage of labour to initiative individualized analgesia. | Support Vector Machine |
| (Salgado Garcia, Indic et al. 2022) | 2022 | To detect self-administration of prescription opioids in patients with acute pain over an extended period using machine learning. | Decision Tree, Logistic Regression, Naive Bayes Classifier, Support Vector Machine, K-Nearest Neighbor |
| (Salgueiro, Basogain et al. 2013) | 2013 | To evaluate the ability of artificial neural networks to predict, on the basis of clinical variables, the response of persons with fibromyalgia syndrome to a standard, 4-week interdisciplinary pain program. | Artificial Neural Network |
| (Sandal, Bach et al. 2021) | 2020 | To investigate the effect of a artificial intelligence self-management application for patients with low back pain. | *Not mentioned* |
| (Sandal, Overas et al. 2020) | 2021 | To investigate the effectiveness an evidence-based, individually tailored self-management support system delivered through an app as an adjunct to usual care for adults with low back pain-related disability. | Case Based Reasoning |
| (Schonnagel, Caffard et al. 2024) | 2024 | To develop a machine-learning algorithm to predict pain outcomes in patients with degenerative lumbar spondylolisthesis undergoing spinal fusion surgery. | Extreme Gradient Boosting, Logistic Regression, Random Forest Classifier, Support Vector Machine |
| (Schwartz, Ward et al. 1997) | 1997 | To predict the relative success of total hip replacement surgery in reducing pain in patients with chronic hip pain. | Neural Network |
| (Seng, Mehdipour et al. 2023) | 2023 | To demonstrate the ability of a natural language processing engine to review clinical notes and accurately identify patients who had persistent postoperative opioid use after major spine surgery. | Natural Language Processing |
| (Shade, Hama et al. 2023) | 2023 | To examine older adults’ preferences for conversational pain management content to incorporate in an interactive artificial intelligent application for pain self-management. | Natural Language Processing |
| (Sharma, Alshehri et al. 2021) | 2021 | To develop a self-management system for patients with chronic lower back pain to help patients monitor their daily activities and provide recommendations regarding activity modification. | Artificial Neural Network |
| (Shieh, Chang et al. 2002) | 2002 | To develop an enhanced patient-controlled analgesia using a hierarchical fuzzy logic control system and to apply it to patients undergoing extracorporeal shock wave lithotripsy | Fuzzy Logic |
| (Shieh, Chang et al. 2007) | 2002 | To propose a novel fuzzy pain demand index derived from the interval of each bolus of patient-controlled analgesia using fuzzy modeling algorithm. | Fuzzy Logic |
| (Shieh, Dai et al. 2007) | 2007 | To compare a fuzzy logic patient-controlled analgesia algorithm was compared with a conventional algorithm, for alfentanil administration in extracorporeal shock-wave lithotripsy | Fuzzy Logic |
| (Shirvalkar, Prosky et al. 2023) | 2023 | To predict intraindividual chronic pain severity scores from neural activity with high sensitivity using machine learning methods using implanted with chronic intracranial electrodes. | Least Absolute Shrinkage and Selection Operator (LASSO) Regression |
| (Sinha, Cheng et al. 2022) | 2022 | To evaluate user retention and engagement with an artificial intelligence–led digital self-management mental health app that is customized for individuals managing mental health symptoms and coexisting chronic pain. | *Not mentioned* |
| (Stojancic, Subramaniam et al. 2023) | 2023 | To determine the feasibility of using the Apple Watch to predict the pain scores in people with sickle cell disease to build and evaluate machine learning algorithms to predict the pain scores of vaso-occlusive crises with the Apple Watch. | Logistic Regression, Gradient Boosting, Random Forest Classifiers |
| (Sun, Li et al. 2023) | 2023 | To describe a novel Interpretable Neural conduct individualized risk assessment of preoperative opioid use to establish patient-centered pain management. | Neural Network Regression |
| (Sun, Kang et al. 2023) | 2023 | To develop prediction models for chronic postsurgical pain after breast cancer surgery using machine learning approaches and evaluate their performance. | Random Forest Classifier, Gradient Boosting Decision Tree, Extreme Gradient Boosting, Logistic Regression |
| (Svendsen, Nicholl et al. 2022) | 2022 | To explore factors influencing embedding, integrating, and sustaining engagement with an artificial intelligence-driven app to support self-management of low back pain as an adjunct to usual care. | *Not mentioned* |
| (Tan, Liu et al. 2021) | 2021 | To evaluate the performance of predictive models for identifying parturients at increased risk of breakthrough pain during labour epidural analgesia. | Random Forest Classifier |
| (Teichmann, Hallmann et al. 2021) | 2021 | To identify dental pain sensation based on cardiorespiratory signals to guide analgesia. | Random Forest Classifier |
| (Thiengwittayaporn, Wattanapreechanon et al. 2023) | 2023 | To develop an interactive mobile application that provides a disease-specific educational background and a structured exercise regimen to patients with painful knee osteoarthritis. | Decision Trees |
| (Tighe, Harle et al. 2015) | 2012 | To develop a model to predict surgical patients that would require a request for a preoperative acute pain service consultation. | Logistic regression, Support Vector Machine, Multilayer Perceptron, K-Nearest Neighbour, Decision Tables, Decision Trees, Random Forest Classifier |
| (Tighe, Lucas et al. 2012) | 2015 | To develop models to forecast which patients will experience moderate to severe postoperative pain in order to guide pain management. | Least Absolute Shrinkage and Selection Operator (LASSO) Regression, Gradient Boosted Decision Tree, Support Vector Machine, Neural Network, K-Nearest Neighbor, Logistic Regression |
| (Tong, Li et al. 2023) | 2023 | To investigate the analgesic effect of artificial intelligence and ultrasound- guided nerve block in total knee arthroplasty. | Recurrent Neural Network |
| (Tsai, Huang et al. 2023) | 2023 | To externally validate a machine learning to predict prolonged postoperative opioid use after total knee arthroplasty. | *Not mentioned* |
| (Tu, Ortiz et al. 2019) | 2019 | To test if pre-treatment resting-state functional connectivity can predict responses to both real and sham acupuncture treatments in chronic low back pain patients. | Multivariate Linear Regression |
| (Verma, Jansen et al. 2022) | 2023 | To investigate to what extent different machine learning methods, applied to two different patient-reported outcome measurement datasets, can predict outcomes among patients with non-specific neck and/or low back pain. | Linear Regression, Random Forest Regression, Stochastic Gradient Descent Regression, Support Vector Regression, Extreme Gradient Boosting Regression |
| (Verma, Bach et al. 2023) | 2022 | To validate machine learning prediction models developed for predicting four pain-related patient-reported outcomes for people with chronic low back pain. | Case-Based Reasoning, Support Vector Regression, and Extreme Gradient Boosting Regression |
| (Visibelli, Peruzzi et al. 2023) | 2023 | To develop machine learning models using data from chronic pain patients treated with cannabis and genotyped for several candidate polymorphic genes to support the therapeutic process and avoiding ineffective results or the occurrence of side effects of medical cannabis. | Extreme Gradient Boosting |
| (Vitzthum, Riviere et al. 2020) | 2020 | To identify clinical risk factors and create a risk score to help identify patients at risk of persistent opioid use and abuse among individuals with cancer-related pain. | Multivariate Logistic Regression |
| (Vuckovic, Gallardo et al. 2018) | 2018 | To create a classifier based on electroencephalography to identify spinal cord injured participants at risk of developing central neuropathic pain by comparing them with patients who had already developed pain and with able bodied controls. | Artificial Neural Network, Support Vector Machine, Linear Discriminant Analysis |
| (Vuong, Utkarsh et al. 2023) | 2023 | To determine the feasibility of remotely monitoring with a consumer wearable during hospitalization for vaso-occlusive crises and up to 30 days after discharge, and to evaluate the accuracy of pain prediction using machine learning models based on physiological parameters measured by a consumer wearable. | Logistic Regression, Random Forest Classifier, Gradient Boosting Model |
| (Wakabayashi, Koide et al. 2021) | 2021 | To establish a predictive model for pain response following radiotherapy using a combination of radiomic and clinical features of spinal metastasis. | Random Forest Classifier |
| (Wang, Kim et al. 2023) | 2023 | To establish an index to monitor the quality of postoperative pain management through machine learning in patients using artificial intelligence patient-controlled analgesia. | *Not mentioned* |
| (Wang, Sun et al. 2021) | 2023 | To develop a model predict the therapeutic outcome of cervical transforaminal lumbar epidural steroid injection in patients with cervical foraminal stenosis. | Convolutional Neural Network |
| (Wang, Liu et al. 2023) | 2023 | To develop a probe based on polarization-sensitive optical coherence tomography (PS-OCT) to enhance epidural anesthesia needle placement using a neural network. | Convolutional Neural Network |
| (Wang, Guo et al. 2023) | 2021 | To evaluate the feasibility and effectiveness of machine learning models for local treatment decision-making to reduce pain in lung cancer patients with bone metastases. | Decision Trees, Support Vector Machine, Bayesian Neural Network |
| (Wang, Li et al. 2023) | 2023 | To develop a prediction model to assess which patients with chronic pain may response to acupuncture treatment using psychological and neurological factors. | Support Vector Machine |
| (Wei, Xu et al. 2022) | 2022 | To evaluate whether the combination of machine learning and amygdala-related functional features could help predict the efficacy of non-steroidal anti-inflammatory drugs in patients with migraine without aura. | Support Vector Machine |
| (Wei, Liao et al. 2022) | 2022 | To elucidate the relationship between analgesic medication outcome and neural activity using electroencephalography and to establish a machine learning model for early prediction of the medication responses from electroencephalography. | K-Nearest Neighbors |
| (Wilson, Colebaugh et al. 2022) | 2022 | To evaluate a clustering method within a perioperative cohort undergoing breast surgery to investigate differences in postsurgical pain outcomes amongst patients to aid in treatment planning. | Nearest Centroid Model |
| (Wirries, Geiger et al. 2021) | 2021 | To determine whether artificial intelligence approaches can be used to predict treatment (including spinal infiltrations, physiotherapy, and psychoeducation) efficiency in patients with back pain. | Neural Network, Hierarchical Clustering |
| (Xu, Xie et al. 2022) | 2022 | To determine whether metabolic patterns in the neuronal pain network might predict acupuncture therapy responses in patients with primary dysmenorrhea using a machine-learning-based multivariate pattern analysis on positron emission tomography data. | Linear Support Vector Regression |
| (Yan, Liu et al. 2023) | 2023 | To construct and validate machine learning models for the early prediction of chronic post-surgical pain among patients undergoing total knee arthroplasty. | Decision Tree, K-Nearest Neighbor, Support Vector Machine; Random Forest Classifier |
| (Yang, Ku et al. 2013) | 2013 | To develop an integrated information system for pain management to provide real-time information for medical staff and offer more efficient and convenient pain control for both in hospital and at-home patients. | Decision Tree |
| (Yen, Ogink et al. 2022) | 2022 | To investigate the generalizability of a machine learning model to predict the risk of prolonged opioid prescription after surgery for lumbar disc herniation. | *Not mentioned* |
| (Zhang, Zhao et al. 2021). | 2023 | To develop models for predicting long-term opioid use in patients after elective spine surgery using preoperative risk factors and 30-day postoperative opioid prescribing patterns. | Logistic Regression, Least Absolute Shrinkage and Selection Operator (LASSO) Regression, Support Vector Machine, Random Forest Classifier, Stochastic Gradient Boosting, Convolutional Neural Network |
| (Zhang, Fatemi et al. 2020) | 2021 | To propose a deep learning model predict postoperative pain in a short period after an operation utilizing dynamic patient data recorded in existing widely utilized equipment (e.g., anesthesia monitor). | Convolutional Neural Network, Graph Transforming Network, Logistic Regression, Support Vector Machine, K-Nearest Neighbor, Decision Tree, Random Forest Classifier, Extreme Gradient Boosting |
| (Zhang, Zhao et al. 2023) | 2020 | To study the analgesic effect of pudendal nerve block on obstetrics and gynecology under the guidance of ultrasound image based on am optimized convolutional neural network algorithm. | Neural Network |
| (Zhu, Niu et al. 2022) | 2022 | To explore the effect of artificial intelligence technology combined with ultrasound-guided needle knife intervention in the treatment of plantar fasciitis on pain, fascia thickness, and ankle and foot function. | Computer Vision |
| (Zmudzki and Smeets 2023) | 2023 | To develop a multidimensional machine learning based upon clinically relevant domains including activity/disability, pain, fatigue, coping and quality of life to prognosticate patients that would respond to an interdisciplinary multimodal pain treatment program. | Decision trees, Support Vector Machines, Logistic Regression, K-Nearest Neighbors, Naïve-Bayes Classifier, Neural Networks |
